# MicroRNA-29a-5p contributes to neuroinflammation through TLR7

**DOI:** 10.1186/s12974-025-03680-4

**Published:** 2026-01-09

**Authors:** Hugo McGurran, Eugenio Graceffo, Victor Kumbol, Marina Jendrach, Lukas Hinkelmann, Mariam Brehm, Leandre Ravatt, Christina Krüger, Thomas Wallach, Alexander Haake, Susanne Wegmann, Frank L. Heppner, Markus Schülke, Seija Lehnardt

**Affiliations:** 1 Neuroscience Research Center, Charité – Universitätsmedizin Berlin, Corporate Member of Freie Universität Berlin and Humboldt-Universität zu Berlin, and Berlin Institute of Health, Berlin, 10117 Germany; 2 Einstein Center for Neurosciences Berlin, Charité – Universitätsmedizin Berlin, Berlin, 10117 Germany; 3 Department of Neuropediatrics, Charité – Universitätsmedizin Berlin, Corporate Member of Freie Universität Berlin and Humboldt-Universität zu Berlin, and Berlin Institute of Health, Berlin, 13353 Germany; 4 Department of Neuropathology, Charité – Universitätsmedizin Berlin, Corporate member of Freie Universität Berlin and Humboldt-Universität zu Berlin, and Berlin Institute of Health, Berlin, 10117 Germany; 5 German Center for Neurodegenerative Diseases (DZNE) within the Helmholtz Association, Berlin, 10117 Germany; 6 Department of Neurology, Charité – Universitätsmedizin Berlin, Corporate Member of Freie Universität Berlin and Humboldt-Universität zu Berlin, and Berlin Institute of Health, Berlin, 10117 Germany

**Keywords:** MicroRNA, Microglia, Neuroinflammation, Alzheimer’s disease, Toll-like receptors, Cytokine expression

## Abstract

MicroRNAs (miRNAs) canonically regulate post-transcriptional gene expression, but they can also serve as ligands for Toll-like receptors (TLRs). These receptors and their associated signalling pathways contribute to inflammatory responses involved in various central nervous system (CNS) diseases, including Alzheimer’s disease (AD). Here, we investigated the effects of extracellularly delivered miRNA in the context of neuroinflammation. We identified several miRNAs specifically dysregulated in AD and/or neuroinflammatory states, which directly activate the single-stranded RNA sensors mouse TLR7 and human TLR7/8. Among them, extracellular miR-29a-5p induced cytokine and chemokine release from murine primary microglia, altered expression of TLR signalling elements, and enhanced Aβ phagocytosis. Furthermore, this miRNA induced neuronal injury dependent on microglial TLR7 expression, but also in a cell-autonomous fashion, in vitro. Intrathecal injection of miR-29a-5p into mice led to microglial accumulation and neuronal injury in the cerebral cortex through TLR7 after 3 days. Brains of wild-type and APP/PS1 mice, an established AD mouse model, treated with multiple intrathecal miR-29a-5p injections over 120 days exhibited changes in cytokine/chemokine expression and neuronal injury. RNAseq analysis of the cerebral cortex of both miRNA-treated genotypes revealed downregulation of MAPK-associated pathways.

Our study establishes AD-associated miRNAs such as miR-29a-5p as TLR7 agonists and signalling molecules for microglia, thereby altering the neuroinflammatory response.

**Supplementary Information:**

The online version contains supplementary material available at 10.1186/s12974-025-03680-4.

## Introduction

MicroRNAs (miRNAs) are fundamental regulators of gene expression that control diverse cellular pathways through mRNA degradation and translational repression. MiRNA dysregulation is associated with a wide range of diseases including cancer [1], cardiovascular [2], autoimmune [3], and neurological disease [4]. Consequently, miRNA dysregulation is intensely discussed with respect to identifying potential therapeutic or diagnostic targets for such diseases, including Alzheimer’s disease (AD) [5]. MiRNAs such as miR-120-5p, miR-320a, miR-204-5p, miR-146a/b-5p, and miR-29, among many others, are specifically dysregulated in AD patients [6–9]. This coincides with the recent discovery that, beyond regulating post-transcriptional gene expression, miRNAs act as ligands for innate immune receptors such as the single-stranded (ss) RNA-sensing Toll-like receptors (TLRs) 7 and 8 [10–13], though this miRNA function remains poorly understood.

TLRs form an essential part of the innate immune system, providing a first line of defence against pathogens. These receptors are predominantly expressed by immune cells including microglia but also certain non-immune cells, such as neurons and endothelial cells [14, 15]. TLR7 detects fragments of viral ssRNA and drives an inflammatory response upon activation. In contrast to other TLRs such as TLR2 and TLR4, which have long been associated with AD and investigated in various AD mouse models [16–18], TLR7 has been less in the spotlight. However, TLR7 expression is increased in AD patients and cerebrospinal fluid (CSF) from AD patients contains increased amounts of *let-7b*, a miRNA ligand of TLR7, suggesting a role for this receptor in AD pathology [12]. Furthermore, TLR7 signalling has been found to affect molecular and cellular processes involved in autophagy [19], cognitive dysfunction [20], apoptosis [21], and neuroinflammation [22].

In principle, inflammation is a highly dynamic process that in the central nervous system (CNS) is primarily driven by microglia and has been established as one of the earliest pathological changes and hallmark in AD [23]. Therefore, modulating neuroinflammation through TLR-associated pathways has been discussed as potential therapeutic options in AD, with multiple clinical trials now assessing its potential [24, 25]. However, inflammation has conflicting roles in AD. For instance, microglial activity, especially in early disease stages, can be beneficial by assisting with the removal of toxic amyloid beta (Aβ) protein, but over time, this inflammatory response can become deleterious, with excess cytokine release causing neurotoxicity and even propagating Aβ pathology [23, 26].

Here, we demonstrate that multiple AD- and/or neuroinflammation-associated miRNAs can act as endogenous ligands for TLR7/8. Among these miRNAs, extracellularly delivered miR-29a-5p potently activated microglia, induced cytokine/chemokine release, and improved phagocytosis of Aβ in vitro*.* Furthermore, miR-29a-5p reduced neuronal viability in the presence of microglia. Intrathecal injection of miR-29a-5p into APP/PS1 mice, a mouse model for AD, led to alterations in cytokine/chemokine expression, downregulation of the MAPK pathway, and resulted in neuronal injury. The observed effects, both in vitro and in vivo, required functional TLR7.

## Methods

### Literature search and *Braindead* analysis

A literature search on Pubmed was performed for peer-reviewed articles using the keywords “microrna microglia neuroinflammation” and “microrna microglia alzheimer”. All associated miRNAs were analysed using the machine-learning software *Braindead* (http://rna.informatik.uni-freiburg.de/ [27]) to determine the potential binding capacity of these miRNAs to TLR7/8. A cut-off value of ≥ 85% was placed to ensure high likelihood of binding. As we sought to identify novel TLR7-activating miRNAs associated with inflammation and AD, known TLR7-activating miRNAs were removed resulting in a final panel of 12 miRNAs (Table 1).

**Table 1:** MiRNA sequences and respective braindead scores. All MiRNAs have 5′ phosphorylation and phosphorothioate bonds in every base

miRNA	Braindead score	Sequence	miRbase ID
hsa-miR-153-5p	0.961	GUCAUUUUUGUGACGUUGCAGCU	MIMAT0026480
hsa-miR-9-5p	0.95	UCUUUGGUUAUCUAGCUGUAUGA	MIMAT0000441
hsa-miR-152-5p	0.938	UAGGUUCUGUGAUACACUCCGACU	MIMAT0026479
hsa-miR-124-5p	0.921	CGUGUUCACAGCGGACCUUGAU	MIMAT0004591
hsa-miR-409-5p	0.904	AGGUUACCCGAGCAACUUUGCAU	MIMAT0001638
hsa-miR-223-5p	0.893	CGUGUAUUUGACAAGCUGAGUUG	MIMAT0004570
hsa-miR-154-5p	0.89	UAGGUUAUCCGUGUUGCCUUCG	MIMAT0000452
hsa-miR-223-3p	0.877	UGUCAGUUUGUCAAAUACCCCA	MIMAT0000280
hsa-miR-29a-5p	0.876	ACUGAUUUCUUUUGGUGUUCAG	MIMAT0004503
mmu-miR-107-5p	0.87	AGCUUCUUUACAGUGUUGCCUUG	MIMAT0017048
hsa-miR-21a-5p	0.855	UAGCUUAUCAGACUGAUGUUGA	MIMAT0000076
hsa-miR-15a-5p	0.85	UAGCAGCACAUAAUGGUUUGUG	MIMAT0000068
hsa-miR-103a-2-5p	0.538	AGCUUCUUUACAGUGCUGCCUUG	MIMAT0009196
Mut. oligo	-	UGAGGUAGAAGGAUAUAAGGAU	-

### Mice

C57BL/6 (WT), *Tlr7*^−/−^, and APP/PS1 mice were bred at the FEM, Charité-Universitätsmedizin Berlin, Germany. *Tlr7*^−/−^ mice were generously provided by S. Akira (Osaka University, Osaka, Japan). Animals were maintained according to the guidelines of the committee for animal care. All animal procedures were approved by the Landesamt für Gesundheit und Soziales (LAGeSo) Berlin, Germany.

### MiRNA oligoribonucleotides

MiRNA oligoribonucleotides with 5′ phosphorylation and phosphorothioate bonds in every base were synthesised by Integrated DNA Technologies. The sequence for all miRNAs used can be found in Table 1. The control oligonucleotide (mut. oligo) is a mutated sequence of *let-7b* with reduced GU content by a six nucleotide exchange in the GU-rich core and has no known homology to any sequence in mouse or human [12]. For all experiments with microglia or HEK TLR reporter cells, miRNAs were complexed and transfected with LyoVec (Invivogen, #lyec-1), which improves uptake and reduces degradation of oligonucleotides [28], unless otherwise indicated. Enriched neuronal cultures were exposed to uncomplexed miRNAs as they do not require transfection for miRNA uptake, as previously described [12, 29]. Details for other chemicals including TLR agonists are provided in Table S1.

### Aβ and tau preparation

HFIP-treated amyloid-beta (Aβ)1–42 (Bachem, 4090148) was reconstituted to 2.5 mM in dimethyl sulfoxide (DMSO). The reconstituted Aβ was diluted to a working concentration of 100 µM in PBS and left at 4 °C overnight prior to experimentation to promote oligomerisation [30–32]. Soluble full length human tau in buffer (25 mM HEPES, 10 mM NaCl, pH 7.4, 1 mM DTT) was produced in *E. coli* BL21 Star™ (Invitrogen) cells as previously described [33, 34] and diluted in PBS to indicated concentration. Both proteins were added directly to cell culture without complexation. Biochemical composition of Aβ and tau were not analysed prior to application in vitro, and protein aggregation, at least in part, cannot be ruled out.

### HEK-blue TLR reporter cells

HEK-Blue™ Secreted Embryonic Alkaline Phosphatase (SEAP) cells (Invivogen, Table S1) expressing either human or mouse TLR7 or TLR8, along with their parental control lines (Null1-k, Null2-k, Null1, and Null1V) were cultured in Dulbecco’s Modified Eagle Medium (DMEM, Gibco, #41965062) supplemented with 10% heat-inactivated fetal calf serum (FCS, Gibco #10082–147), penicillin/streptomycin (100 U/mL; 100 µg/mL, Gibco #15140–122). For SEAP detection, 30,000 cells were seeded onto 96-well plates, and the following day, media was changed to 90% HEK-Blue detection reagent (Invivogen, #hb-det2) and 10% DMEM. Cells were incubated for 24 h with indicated miRNAs (10 µg/mL). LyoVec and mut. oligo served as negative controls. TNF (NF-κB/AP-1 promoter activation), loxoribine (TLR7), and R848 (TLR7/8) served as positive controls (Table S1). Conditions were performed in triplicate and absorbance measured at 655 nM using a SpectraMax iD3 (Molecular Devices). SEAP detection experiments were only performed on cells with < 20 passages to ensure cell line stability. Additionally, cells were further treated with Blasticidin, Zeocin, and Normocin according to manufacturer’s instructions to prevent bacterial, fungal, and mycoplasma growth.

### Primary cell culture

#### Neurons

Primary neuronal cultures were generated from forebrains of embryonic day E17 C57BL/6 mice, as previously described [11]. In brief, meninges, superficial blood vessels, and cerebellum were removed from the cortices. Cortices were then manually homogenised and incubated with 2.5% trypsin (Gibco #15090046) for 20 min at 37 °C and quenched with FCS. The tissue was then treated with 100 µL DNase (Roche #1284932001) and washed thoroughly. The cell suspension was then centrifuged at 17 RCF, and the resulting supernatant was centrifuged at 264 RCF. The pellet was resuspended in neurobasal media (Gibco #21103049), and 5 × 10^5^ cells were added to PDL-coated glass coverslips in a 24-well plate. A half media change was performed the following day. Cells were then incubated at 37 °C in humidified air with 5% (*v/v*) CO_2_ for 3 d before experimentation.

#### Microglia

Primary microglia were generated from the cortex of P1-4 C57BL/6 or *Tlr7*^−/−^mice as previously described [11]. Briefly, the brain was removed. Cortices were manually homogenised and incubated with 2.5% trypsin for 25 min at 37 °C. Tissue was treated with 100 µL DNase and centrifuged at 264 RCF for 5 min. The pellet was resuspended in DMEM, passed through a 70 μm strainer, and added into T75 culture flasks. A full media change was performed the following day. Cells were then incubated for 10–14 d at 37 °C in humidified air with 5% (*v*/*v*) CO_2_. Microglia were removed by shaking on an orbital shaker at 200 rpm for 20 min.

#### Co-cultures of neurons and microglia

To generate co-cultures, neurons and microglia were prepared as described above. Once neurons had been in culture for 3 d, half of the neuronal media was removed and replaced with DMEM containing 60,000 microglia for an approximate ratio of 1:8 microglia to neurons. Experiments were conducted the following day. For conditioned media experiments, 60,000 microglia in 500 µL DMEM were stimulated with indicated condition for 24 h. Once neurons had been in culture for 3 d, half the neuronal media was removed and replaced with 250 µL of the conditioned DMEM for 5 d.

### Tumour necrosis factor Enzyme-Linked immunosorbent assay (ELISA)

Thirty thousand primary mouse microglia were incubated with free or LyoVec-complexed miRNAs at indicated concentration, loxoribine (1 mM) or LPS (100 ng/mL) for the indicated duration. Supernatants of treated cells were analysed with a commercial TNF ELISA kit according to manufacturer’s instructions (Invitrogen, #88732488).

### Multiplex immune and Aβ protein assays

For analysis of cyto- and chemokine expression, 30,000 primary mouse microglia were incubated with miR-29a-5p (10 µg/mL), mut. oligo (10 µg/mL), loxoribine (1 mM), or LPS (100 ng/mL) for 24 h. Supernatants were removed and stored at -70 °C until use. Tissue samples from WT and APP/PS1 mice were homogenised and lysed in RIPA buffer (Pierce, #89900) using the Precellys Evolution Touch homogeniser (Bertin Technologies). Lysates were centrifuged at 14,000 rpm for 20 min, and the supernatant was removed. A BCA assay (Pierce, #23225) was used to normalise total protein concentration in all samples. Samples were analysed using a custom U-plex multiplex kit according to manufacturer’s instructions (Meso Scale Diagnostics). For analysis of Aβ38, Aβ40, and Aβ42, tissue samples were homogenised followed by a three-step extraction first in TBS, then TX, and then in SDS to extract Aβ with different solubility. The TBS fraction containing soluble Aβ (undiluted) and the SDS fraction containing insoluble/plaque-associated Aβ (diluted at 1:500 with DTBS) were analysed with the V-PLEX Aβ Peptide Panel 1 (6E10) Kit (Meso Scale Discovery, #K15200E-1) using a Tecan Infinite^®^ 200 Pro (Tecan Life Sciences) and normalised on the protein content, which was measured with a BCA assay (BCA Protein Assay Kit (ThermoFisher, #23227). Multiplex assays were analysed using a Meso Sector S 600MM device.

### Reverse transcription quantitative polymerase chain reaction (RT-qPCR)

Primary microglia or neurons were stimulated for the indicated time with miR-29a-5p (10 µg/mL), LPS (100 ng/mL), or loxoribine (1 mM). RNA was collected as per manufacturer’s instructions using a commercial kit (Qiagen, #74104). cDNA was synthesised using M-MLV reverse transcriptase (Promega, M1701), and SYBR-green qPCR performed. For quantification of *miR-29a-5p* expression in primary cells, microglia or neurons were stimulated for 24 h with miR-29a-5p mimic (10 µg/mL) or loxoribine (1 mM). For analysis of WT and APP/PS1 tissue samples, cortex tissue was homogenised using the Precellys Evolution Touch homogeniser (Bertin Technologies) in lysis buffer. RNA was collected as per manufacturer’s instruction using a commercial kit (Qiagen, #217084). cDNA was synthesised, and qPCR performed using a commercial kit (Qiagen, #339320). miR-29a-5p expression was normalised to endogenous control miR-103-3p. SYBR-green qPCR was performed using the StepOnePlus RT-qPCR system (Applied Biosystems). All data are expressed using 2^–∆∆Ct^. Conditions were performed in triplicate and averaged. Primers used are listed in Table S1.

### Phagocytosis assay

5-FAM-amyloid-beta (Aβ)1–42 (Bachem, 4090151) was reconstituted to 2.5 mM in DMSO and subsequently diluted to a working concentration of 100 µM in PBS and left at 4 °C overnight prior to experimentation. To measure phagocytosis, 30,000 microglia were plated in a 96-well plate on coverslips and the following day, microglia were treated with miRNA or control alongside 500 nM of 5-FAM-Aβ1–42 for 24 h. Cells were then thoroughly washed and fixed in 4% PFA for 20 min before immunostaining with Iba1 antibody. Phagocytic capacity of stimulated cells was assessed by calculating the percent area of Aβ1–42 within Iba1-positive cells and normalised to the unstimulated control, as described previously [35].

### Immunocytochemistry and immunohistochemistry

Cells grown on cover slips were thoroughly washed and fixed in 4% PFA for 20 min. Cells were stained with indicated primary antibodies (Table S1) in staining buffer (PBS, 2% NGS, 0.2% Triton-X) overnight at 4 °C. Cells were washed and incubated with secondary antibodies (Table S1) for 1 h at room temperature. Coverslips were then sealed with immumount (Epredia, 9990402). For TUNEL apoptosis assays (Roche, 11684795910, 12156792910), experiments were conducted following manufacturer’s instructions. For tissue staining, brains were fixed in 4% PFA and cut coronally at 14 μm thickness and thaw-mounted on glass slides. Prior to staining, tissue was treated with 4% PFA for 15 min, thoroughly washed, then treated with blocking buffer (PBS, 5% NGS, 0.2% Triton-X) for 1–3 h. Sections were stained with indicated primary antibodies in staining buffer overnight at 4 °C. Sections were washed and incubated with secondary antibodies for 1 h at room temperature and sealed with immumount.

### Microscopy and imaging

For imaging of cell cultures stained with TUNEL assay or NeuN and MAP2 immunostaining, two coverslips per condition were analysed. Six images of each coverslip at 40× magnification were taken on an Olympus IX81 microscope. Data was normalised to unstimulated control. For imaging and quantification of NeuN and Iba1 immunostaining in the protocol describing intrathecal injection into mice after 3 d (see below), images of both the left and right hemisphere were taken at approximately an interaural distance of 1.9 mm on an Olympus IX81 microscope. Quantification was performed by two independent examiners and data were pooled for analysis. Imaging for neurofilament and MAP2 immunostaining 3 d post-injection was performed by taking 3 images of both the left and right hemisphere at approximately an interaural distance of 1.9 mm of the retrosplenial area, cingulum bundle, and dentate gyrus at 10× magnification. Imaging for GFAP immunostaining was performed using a Nikon widefield ti2 microscope at 10× magnification taking a stitched image of the entire left and right hippocampus at approximately interaural distance 1.9 mm. Imaging for neurofilament, MAP2, and caspase-3 immunostaining in long-term (3 monthly injections) WT and APP/PS1 experiments, 6 images of the cortex were taken at interaural distance of 1.9 mm on an Olympus IX81 microscope. Imaging of Iba1 and Aβ 4G8 immunostaining for APP/PS1 experiments was performed using a Nikon Widefield ti2 at 10× magnification taking a stitched image of the entire hemisphere of which the entire cortex of 5 sections per mouse was analysed at approximately interaural distances 6.6, 5.3, 3.9, 2.1, and 1.9 mm. Examiners were blinded, and all imaging was analysed with FIJI [36].

### Analysis of miR-29a-5p uptake by microglia

Primary microglia were prepared as described above, and 1 × 10^5^ microglia were seeded onto PDL-coated coverslips. Cells were incubated with the endosomal marker pHrodo Red Dextran (20 µg/mL) and Alexa-488-labelled miR-29a-5p (10 µg/mL), and incubated at 37 °C for 4 h. Microglia were then washed with PBS, fixed with 4% PFA for 20 min, and stained with DAPI. Subsequently, cells were imaged on a Nikon CSU-X1 Spinning Disk Confocal microscope. Image stacks (0.5 μm step size) were acquired with a 100X objective. The fluorescence intensity along linear ROIs traversing endosomes were analysed in FIJI to confirm colocalization.

### Intrathecal injection into mice

Intrathecal injection into mice was performed, as described previously [12, 37]. Briefly, 10 µg of miRNA or loxoribine (136 µg) in 40 µL water, or water alone as vehicle control was injected into the intrathecal space. For intrathecal injection into WT and *Tlr7*^−/−^ mice, 3 d post injection, mice were perfused with 4% PFA, and the whole brain was placed in a 30% sucrose solution, then cut coronally at 14 μm thickness, and thaw-mounted onto glass slides for analysis. For WT and APP/PS1 mice, the injection was carried out as described above every 30 d for a total of 3 injections. The locked nucleic acid (LNA) inhibitor and control inhibitor (Qiagen, YI04101188; YI00199006) were injected 16 h prior to miRNA injection. Mice were perfused with 4% PFA 30 d after the final injection. The brain was removed, and one hemisphere was cryoprotected in 30% sucrose solution, then cut coronally at 14 μm thickness, and thaw-mounted onto glass slides. The other hemisphere was snap-frozen in liquid nitrogen and kept at -70 °C for RNA and protein analysis.

### Library preparation and sequencing

RNA was isolated from snap-frozen left hemispheres of all mice using a commercial kit according to manufacturer’s instructions (Qiagen, #74104). A minimum of 500 ng total RNA from each sample was sent to the Berlin Institute of Health Genomics facility using the Novaseq X plus platform generating 100 million 100 bp reads. Differentially expressed genes and mouse characteristics can be found in Table S2.

### Bioinformatic analyses of RNA-seq data sets

Sequence quality was assessed using FastQC v0.11.8 (www.bioinformatics.babraham.ac.uk/projects/fastqc, accessed on 4 Oct 2024) and MultiQC v1.6 [38]. Reads were mapped to the EMBL mus musculus genome assembly GRCm39 (mm39, release 112) using the splice-aware aligner STAR v2.7.10a [39]. BAM files were sorted and indexed using SAMtools v1.9 [40]. StringTie v2.1.7 was used to generate the gene count matrix [41]. PCA, clustering and differential expression analysis were performed in R v4.3.2 with the DESeq2 v1.42.0 package and used to remove outliers that did not cluster to condition or genotype (outlier genotype: APP/PS1, condition: inhibitor + miR-29a-5p, *n* = 1. For all other groups and conditions, *n* = 3). For visualising purposes, LFC shrinkage using “apeglm” was performed [42]. Unless otherwise stated, a Padj (FDR) value < 0.1 was considered statistically significant in DE analysis. GO enrichment Analysis of Biological Processes was performed using the *enrichGO* function of the clusterProfiler v4.10.0 package.

### Statistics

All data are expressed as the mean ± SEM. Normality was tested with the Shapiro-Wilk test. Statistical analyses comparing two groups were tested with Student’s *t*-test or by multiple *t*-tests with FDR (two-stage step-up method), as indicated. For comparison of multiple groups, a one-way ANOVA followed by Dunnett’s or Sidak’s test to account for multiple comparison was used. For experiments including WT, *Tlr7*^−/−^, and APP/PS1 mice a two-way ANOVA followed by Tukey’s test was performed. Statistics were calculated in Graphpad Prism 9.0 (Dotmatics).

## Results

### Identifying AD- and neuroinflammation-associated MiRNAs as endogenous TLR7/8 ligands

SsRNA fragments represent the natural ligands for TLR7 and TLR8. Also, certain miRNAs have been shown to directly activate TLR7 and TLR8 in a sequence-dependent manner [10, 27, 43]. We aimed to identify miRNAs associated with AD and/or neuroinflammation to evaluate their potential as signalling molecules for TLR7/8. To this end, we performed a literature search for relevant miRNAs whose expression is specifically dysregulated in AD and/or in neuroinflammatory states. We then leveraged the machine-learning software *Braindead*, which predicts the potential for small RNA molecules to serve as ligands for TLR7/8. Although this algorithm was originally trained on data from mouse microglia, it is capable of accurately predicting small RNAs that bind to both mouse and human TLR7/8 [27]. We identified a total of 12 miRNAs that had a high probability (≥ 85%) of being able to bind to TLR7/8 (see Table 1). An additional miRNA, hsa-miR-103a-2-5p as the closest human equivalent of mmu-miR-107-5p was included in the resulting candidate list, as this miRNA met the threshold in the *Braindead* analysis but did not have an exact human homologue. All other candidate miRNAs have identical human and mouse sequences.

**Fig. 1:**
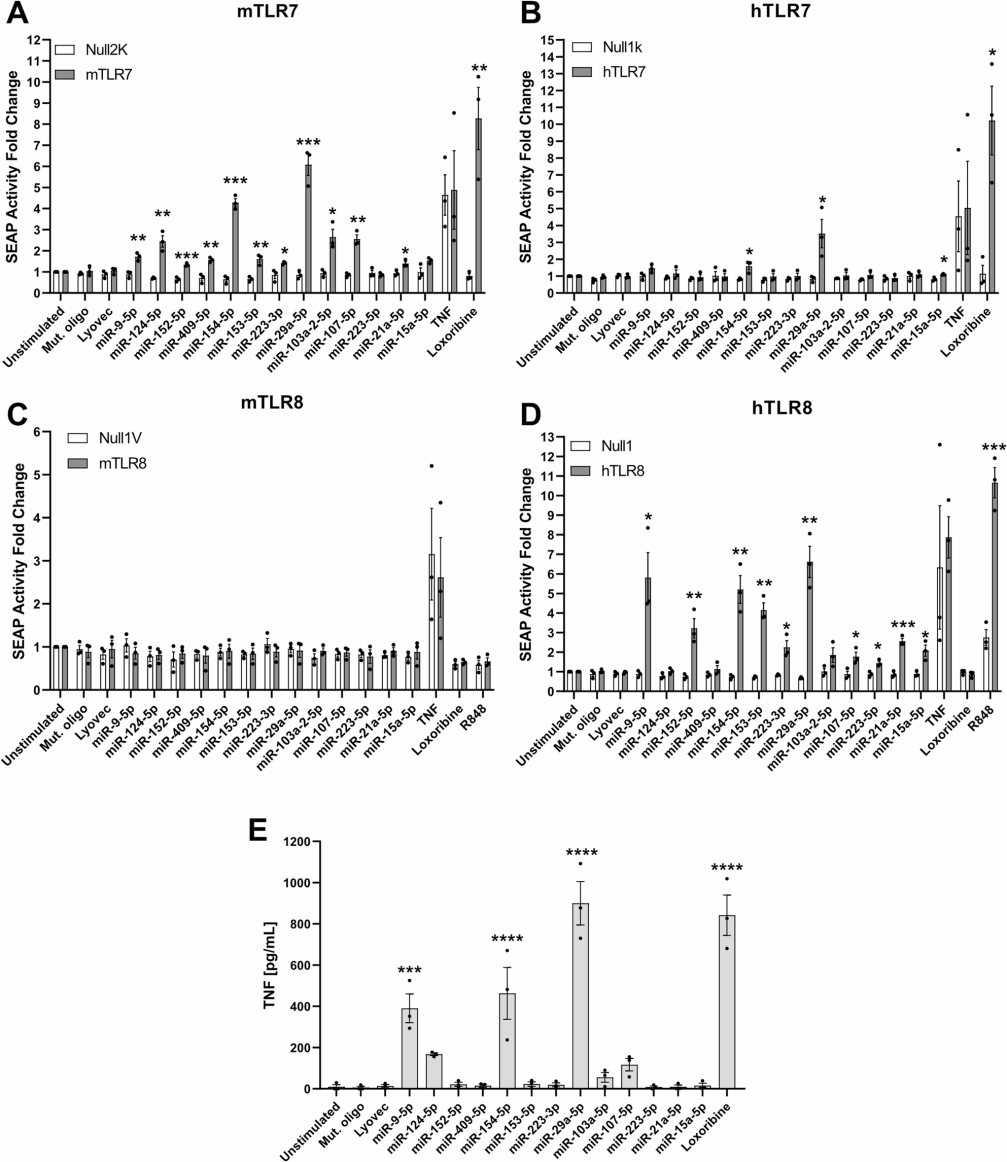
AD- and neuroinflammation-associated miRNAs directly activate ssRNA-sensing TLRs. HEK TLR reporter cells expressing **A**) mouse TLR7, **B**) human TLR7, **C**) mouse TLR8, or **D**) human TLR8 were exposed to indicated miRNA (10 µg/mL) for 24 h. Unstimulated condition, mut. oligo (10 µg/mL), and the transfection agent LyoVec served as negative controls. Loxoribine (1 mM), R848 (100 ng/mL), and TNF (100 ng/mL) served as positive control for TLR7, TLR8, and reporter cell activation, respectively. Fold change calculated as change in absorbance from NF-κB-inducible SEAP compared to parental control line for each condition. **E**) C57BL/6 (wild-type, WT) microglia were exposed to indicated miRNA (10 µg/mL) for 24 h. TNF concentrations in the supernatant were assessed by ELISA. Significance determined by multiple *t*-test for HEK TLR cell analysis or one way ANOVA followed by Dunnett’s multiple comparison test for ELISA. *p < 0.05; **p < 0.01; ***p < 0.001; ****p < 0.0001. Error bars represent mean ± SEM. *n* = 3

To validate the in silico results described above, we conducted a screen approach using HEK reporter cells overexpressing either human or mouse TLR7 or TLR8. Supporting the *Braindead* results, 11 out of 13 of the screened miRNAs significantly activated mouse TLR7, with miR-29a-5p comparatively inducing the strongest receptor response, and with only miR-223-5p and miR-15a-5p failing to activate the receptor (Fig. 1A). Three miRNAs, namely miR-29a-5p, miR-154-5p, and miR-15a-5p, were able to activate human TLR7 (Fig. 1B), with miR-29a-5p and miR-154-5p activating both mouse and human TLR7. Mouse TLR8 was not activated by any miRNA candidates in extracellular form (Fig. 1C), an effect likely due to the mouse TLR8 receptor’s lack of or altered function in detecting ssRNA compared to the human receptor [44]. Finally, human TLR8 was activated by 10/13 candidates, including miR-29a-5p, with only miR-124-5p, miR-409-5p, and miR-103a-2-5p not doing so (Fig. 1D). A mutated control oligoribonucleotide (mut. oligo, hereafter), previously shown not to activate TLR7 or TLR8 [12], failed to induce a response from mouse or human TLR7 or TLR8, as expected.

To test the functional relevance of the miRNAs identified as activators of TLR7 and TLR8, C57BL/6 (WT) primary mouse microglia, which express all known TLRs including TLR7 and TLR8 [45], were exposed to these synthetic miRNAs in vitro, and TNF release, as an indicator of cellular activation, was assessed by ELISA. From all tested miRNAs, miR-9-5p, miR-154-5p, and miR-29a-5p induced significant TNF release from microglia. MiR-29a-5p in particular induced a TNF response to a similar extent as loxoribine, an established synthetic TLR7 agonist [46] (Fig. 1E).

Taken together, we identified several AD- and neuroinflammation-associated miRNAs as ligands for mouse and human TLR7 and/or human TLR8. Given its potent activation of both mouse and human TLR7, human TLR8, and primary microglia, we subsequently focussed on miR-29a-5p as a potential signalling molecule in the context of neuroinflammation in the following experiments.

### Extracellular miR-29a-5p induces microglial release of inflammatory molecules and alters Aβ phagocytosis through TLR7 in vitro

**Fig. 2:**
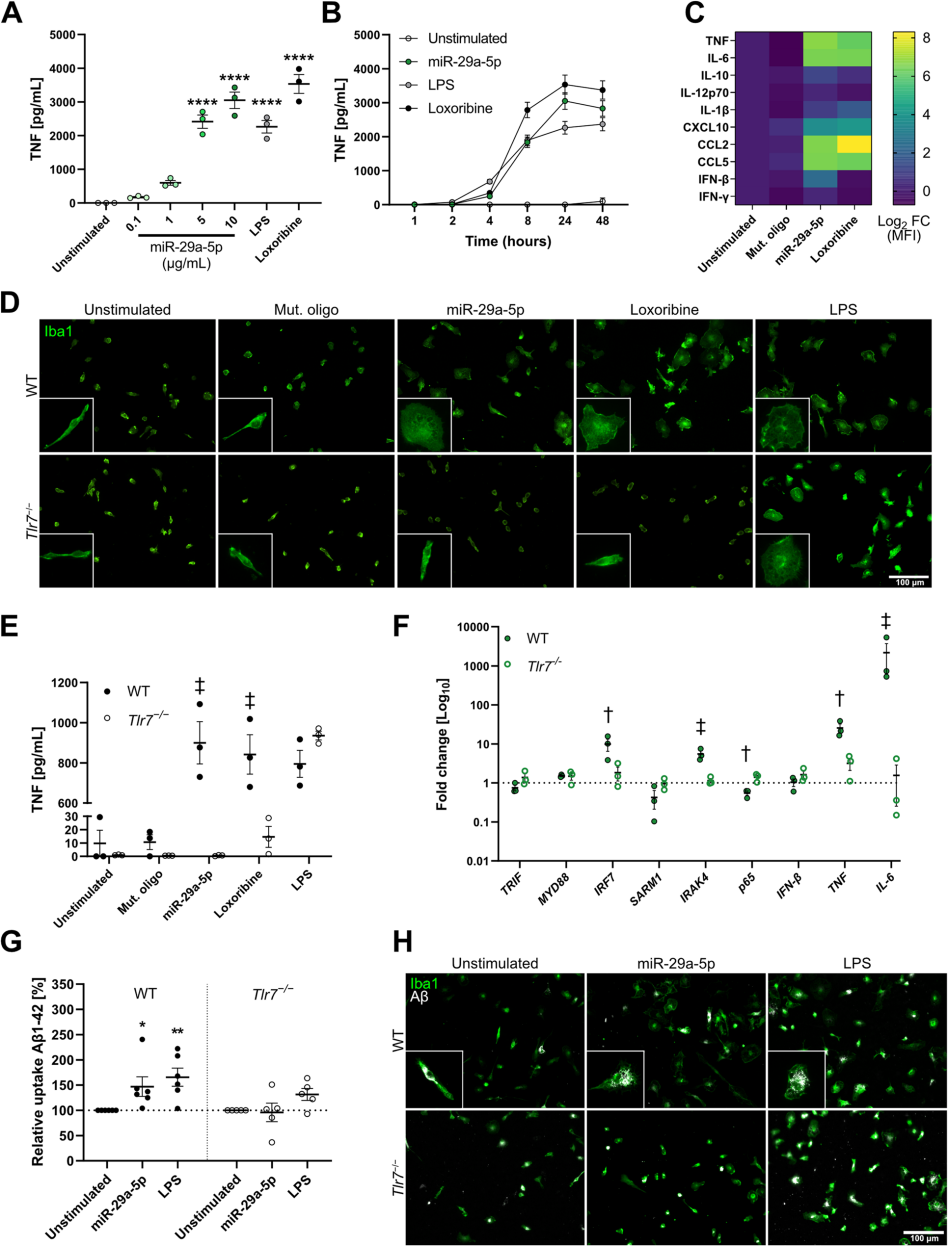
Extracellular miR-29a-5p induces microglial cytokine and chemokine release, as well as Aβ phagocytosis dependent on TLR7. **A**) WT microglia were exposed to indicated miR-29a-5p doses for 24 h, and TNF concentration in the supernatant was measured by ELISA. Loxoribine (1 mM) and LPS (100 ng/mL) served as positive controls. **B**) WT microglia were exposed to 10 μg/mL miR-29a-5p for indicated durations, and TNF concentration in the supernatant was measured by ELISA. Loxoribine (1 mM) and LPS (100 ng/mL) served as positive controls. **C**) Multiplex immunoassay of WT microglia exposed to miR-29a-5p (10 μg/mL) for 24 h. Mut. oligo (10 μg/mL) and loxoribine (1 mM) served as controls for sequence specificity and TLR7 activation, respectively. **D**) Representative images of morphological changes in WT and *Tlr7*^*−/−*^microglia after 24 h of indicated treatments. **E**) WT and vv *Tlr7*^*−/−*^ microglia were exposed to mut. oligo, miR-29a-5p (10 μg/mL), or LPS (100 ng/mL) for 24 h, and TNF concentration in the supernatant was measured by ELISA. **F**) WT and *Tlr7*^*−/−*^microglia were exposed to miR-29a-5p (10 μg/mL) for 24 h. RNA was extracted from cells for analysis of the TLR signalling pathway by RT-qPCR (see Table S1 for primers). **G**) WT (*n* = 6) and *Tlr7*^*−/−*^(n = 5) microglia stimulated with miR-29a-5p (10 μg/mL) or LPS (100 ng/mL) were simultaneously given 5-FAM-tagged Aβ1-42 (500 nM) and assessed for Aβ1-42 uptake after 24 h. **H**) Representative images of WT and *Tlr7*^*−/−*^microglia phagocytosing Aβ1-42. Scale bars represent 100 μm. Significance was determined using either a one-way ANOVA followed by Dunnett’s test **A**, **G**), by unpaired t-tests when comparing WT and *Tlr7*^*−/−*^directly **E**, **G**), or multiple unpaired t-tests by two-stage step-up method F). *p < 0.05; **p < 0.01 to unstimulated condition, †*p* < 0.05; ‡*p* < 0.01 to *Tlr7*^*−/−*^of same condition. Error bars represent mean ± SEM. *n* = 3 (A-F), *n* = 5-8 (**G**)

To further investigate the effect of extracellular miR-29a-5p on microglia, we performed a dose-response analysis in primary wild-type (WT) microglial cultures exposed to miR-29a-5p. We detected significant TNF release after 24 h beginning at 5 µg/mL of miR-29a-5p, with the highest concentration tested (10 µg/mL) inducing a similar response to that of loxoribine and LPS (Fig. 2A). This miR-29a-5p dose induced substantial TNF release after 4 h, with a peak after 24 h. Likewise, LPS and loxoribine induced a peak of TNF release after 24 h (Fig. 2B). MiRNA transfection using LyoVec increased TNF release from microglia by ~ 3-fold compared to usage of uncomplexed miR-29a-5p (Fig. S1A), aligning with a previous study [47]. The inflammatory response of WT microglia exposed to miR-29a-5p after 24 h was further analysed by a multiplex immune assay (Fig. 2C, Table S2). Compared to the unstimulated condition, extracellularly delivered miR-29a-5p induced substantial release of cytokines, including TNF and IL-6, to a similar extent as loxoribine. Both IL-10 and IL-12p70 concentrations were increased after exposure to miR-29a-5p, but not loxoribine, indicating a microglial response specifically induced by miR-29a-5p. MiR-29a-5p exposure also led to the release of chemokines such as CCL2, CCL5, and CXCL10, to a similar extent as loxoribine. None of the tested conditions induced IFN-β or IFN-γ production in microglia. As expected, mut. oligo caused no substantial release of inflammatory molecules from microglia (Fig. 2C). Exposure of microglia to miR-29a-5p led to morphological changes, with miRNA-treated microglia displaying a distinct amoeboid morphology comparable to what is observed in microglia treated with loxoribine or LPS (Fig. 2D). Microglial uptake of extracellularly applied miR-29a-5p by microglia was confirmed when Alexa-488-tagged miR-29a-5p mimic co-localised with the endosomal marker pHrodo Red after 4 h of miRNA exposure (Fig. S1B).

To determine whether the observed miRNA-induced inflammatory response from microglia requires TLR7, *Tlr7*^*−/−*^ microglia were exposed to miR-29a-5p for 24 h. Cells were also incubated with loxoribine as a negative control or LPS as a positive control for microglial activation via TLR4 (Fig. 2E). In contrast to WT cells, *Tlr7*^−/−^ microglia did not release TNF after miR-29a-5p exposure, indicating TLR7 as the responsible receptor mediating miR-29a-5p-induced TNF release from WT microglia. Furthermore, *Tlr7*^−/−^ microglia maintained a ramified state compared to WT microglia exposed to miR-29a-5p (Fig. 2D).

To assess the expression of canonical TLR pathway elements in WT and *Tlr7*^−/−^ microglia exposed to miR-29a-5p, we performed RT-qPCR analysis (Fig. 2F). LPS served as a positive control for induction of the TLR signalling pathway (Fig. S2A). Extracellular miR-29a-5p increased transcription of the regulatory TLR signalling components *IRF7* and *IRAK4*, *p65*, as well as *TNF* and *IL-6* mRNA in WT, but not *Tlr7*^−/−^ microglia. Unlike LPS, miR-29a-5p did not alter *IFN-β* expression (Fig. S2A, B), which is typically induced upon activation of the canonical TLR signalling pathway [48]. Expression of the primary adaptor proteins *MYD88* and *TRIF* was unchanged in response to miRNA stimulation compared to unstimulated control. In WT microglia, the described alterations in TLR signalling started as early as 6 h post-stimulation (Fig. S2B). No such transcriptional changes in the expression of TLR signalling elements were observed in primary WT neurons (Fig. S2C). Exposure to miR-29-5p mimic increased *miR-29a-5p* expression in WT microglia. Loxoribine downregulated its expression, while Aβ had no significant effect (Fig. S2D).

Next, the effect of miR-29a-5p exposure on microglial phagocytic activity in the context of AD was assessed. WT microglia responded to extracellular miR-29a-5p by increasing phagocytic uptake of Aβ1–42 compared to unstimulated control. However, in *Tlr7*^−/−^ microglia, miR-29a-5p did not increase Aβ phagocytosis compared to unstimulated indicating a role for this receptor in miRNA-modulated Aβ phagocytosis (Fig. 2G, H). Of note, baseline phagocytic activity of *Tlr7*^−/−^ microglia appeared to be, although not statistically significant, greater than that of WT microglia (data not shown). Thus, relative phagocytic activity between genotypes were not directly compared (Fig. 2G).

### Extracellular miR-29a-5p induces neuronal injury in the presence of microglia in vitro

**Fig. 3:**
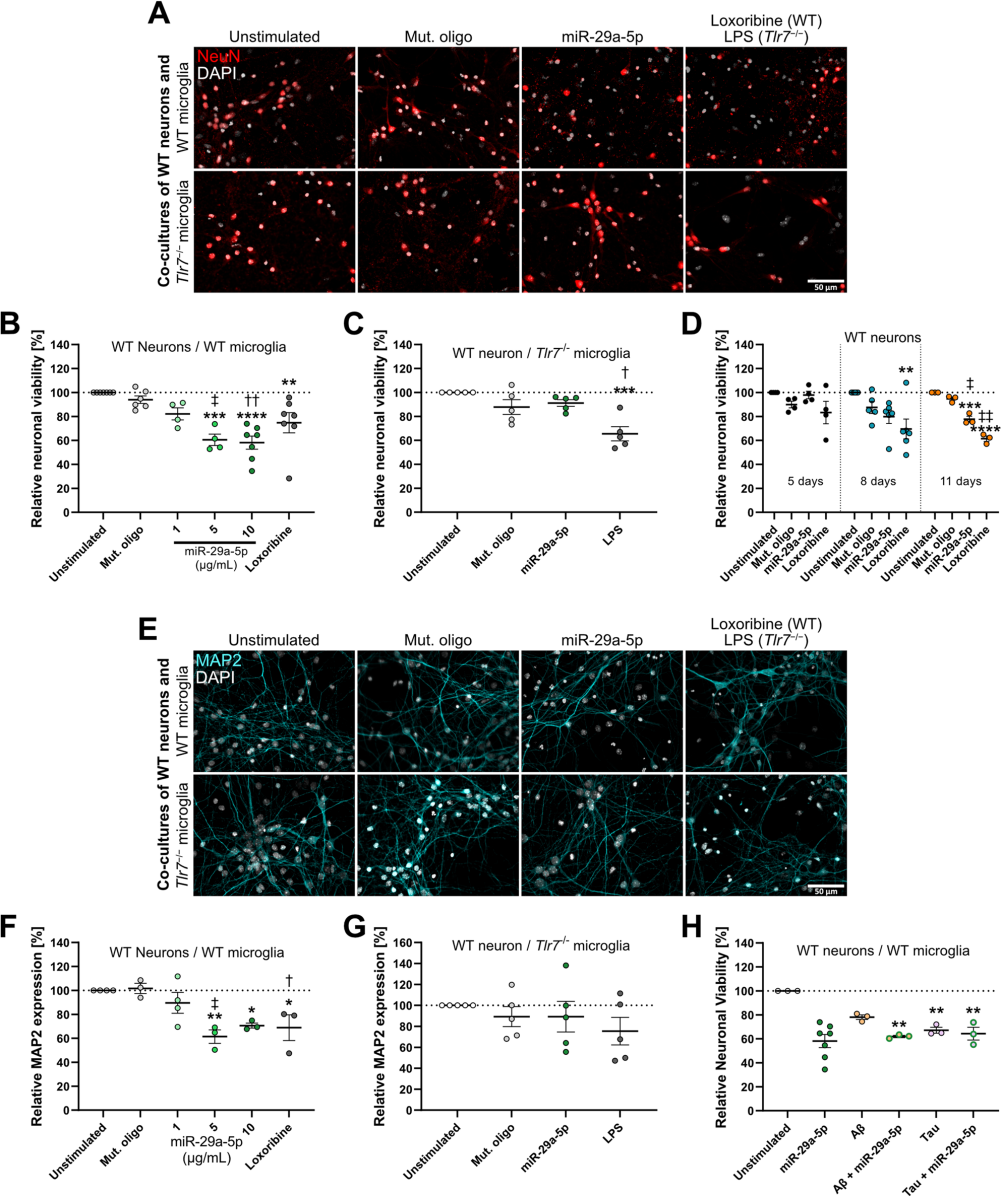
Extracellular miR-29a-5p reduces neuronal viability in vitro. **A**) Representative images of NeuN immunostaining and DAPI staining of co-cultures containing C57BL/6 (WT) neurons with either WT or *Tlr7*^−/−^ microglia after stimulation with indicated agents for 5 d. **B**) Co-cultures of WT neurons with WT, or **C**) *Tlr7*^−/−^ microglia were stimulated with indicated dose or 10 µg/mL of miR-29a-5p for 5 d and immunostained with NeuN antibody to assess neuronal numbers. Mut. oligo (10 µg/mL), loxoribine (1 mM), or LPS (100 ng/mL) served as controls. **D**) WT neurons stimulated with mut. oligo, miR-29a-5p (both 10 µg/mL), or loxoribine (1 mM) for 5, 8, or 11 d, and subsequently immunostained with NeuN antibody to assess neuronal viability. **E**) Representative images of MAP2 immunostaining and DAPI staining of co-cultures containing WT neurons with either WT or *Tlr7*^−/−^ microglia after stimulation with indicated agents for 5 d. **F**) Co-cultures of WT neurons with WT, or **G**) *Tlr7*^−/−^ microglia were stimulated with indicated dose or 10 µg/mL of miR-29a-5p for 5 d and immunostained with MAP2 antibody to assess relative expression. **H**) Co-cultures of WT neurons and WT microglia were treated with Aβ1–42 (10 µM) or Tau (100 nM), miR-29a-5p (10 µg/mL), or both, for 5 d, and subsequently immunostained with NeuN antibody to assess neuronal viability. Dashed line indicates unstimulated condition. Scale bars represent 50 μm. Significance tested with a one-way ANOVA followed by Sidak’s multiple comparison test to unstimulated condition or mut. oligo. *p < 0.05; *p < 0.01; ***p < 0.001; ****p < 0.0001 to unstimulated condition. ^†^*p <* 0.05; ^‡^*p <* 0.01; ^††^*p <* 0.001 to mut. oligo. Error bars represent mean ± SEM. *n* = 3–7.

Small RNA molecules are capable of inducing neuronal injury through microglia [10, 12]. As we had observed that miR-29a-5p is a potent activator of microglia, we assessed neuronal viability after exposure to this miRNA in co-cultures of WT neurons and WT microglia. MiR-29a-5p led to a significant and dose-dependent reduction of neuronal viability, with approximately 40% neuronal loss at the highest dose (10 µg/mL) used over 5 d (Fig. 3A, B). Also, we observed distinct changes in microglial morphology, i.e. increase in amoeboid cell shape, enlarged cell bodies, and cellular extensions, indicating the microglial response to miR-29a-5p exposure (Fig. S3A). Conditioned media from miR-29a-5p-stimulated microglia induced significant neuronal loss after 5 d (Fig. S3B, C). Thus, the reduction in relative neuronal viability after miR-29a-5p treatment was assumed to be due, at least in part, to toxic soluble factors released by microglia upon TLR activation, as previously described [49, 50]. Also, it is possible that the observed neurotoxic effects in co-cultures were due to microglia phagocytosing neurons. In contrast, when WT neurons were co-cultured with *Tlr7*^−/−^ microglia at the highest miR-29a-5p concentration (10 µg/mL), neurotoxic effects observed in co-cultures containing WT microglia were prevented, with no change in neuronal viability compared to control conditions (Fig. 3A, C). Incubation of enriched cortical neurons with miR-29a-5p slightly reduced relative neuronal viability, with significant neuronal loss detected after 11 d (Fig. 3D). Numbers of TUNEL-positive cells were unchanged in these enriched neuronal cultures compared to unstimulated control (Fig. S3D, E). In contrast, loxoribine caused injury in enriched neuronal cultures, as expected from previous studies on cell-autonomous neurotoxicity, which is mediated through neuronal TLR7 [10–12]. *MiR-29a-5p* expression as measured by RT-qPCR was increased in cortical neurons exposed to miR-29a-5p mimic after 24 h. Stimulation with Aβ or loxoribine did not lead to significant effects on *miR-29a-5p* expression in these cells (Fig. S3F).

In line with the observed neurotoxic effects in co-cultures containing WT microglia, extracellular miR-29a-5p significantly reduced numbers of dendrites in the presence of WT microglia (Fig. 3E, F). In contrast, dendrite numbers in co-cultures containing *Tlr7*^−/−^ microglia were not affected by miR-29a-5p treatment (Fig. 3E, G). Given that extracellular miR-29a-5p induced substantial loss of neurons and dendrites in the presence of microglia, we questioned whether the inclusion of AD-associated proteins such as Aβ and tau would affect these miRNA-induced neurotoxic effects. In co-cultures of WT neurons and microglia, tau induced significant neurotoxic effects, while Aβ1–42 elicited a response with a similar trend, although not reaching statistical significance (*p* = 0.09), over 5 d. Notably, although miR-29a-5p increased Aβ phagocytosis at low protein concentrations (see Fig. 2G) combinatorial treatment of high Aβ or tau concentration with miR-29a-5p did not alter neurotoxic effects beyond that of miR-29a-5p or Aβ/tau protein treatment alone (Fig. 3H).

### Intrathecal miR-29a-5p causes microglial accumulation and neuronal loss in the murine cerebral cortex

**Fig. 4:**
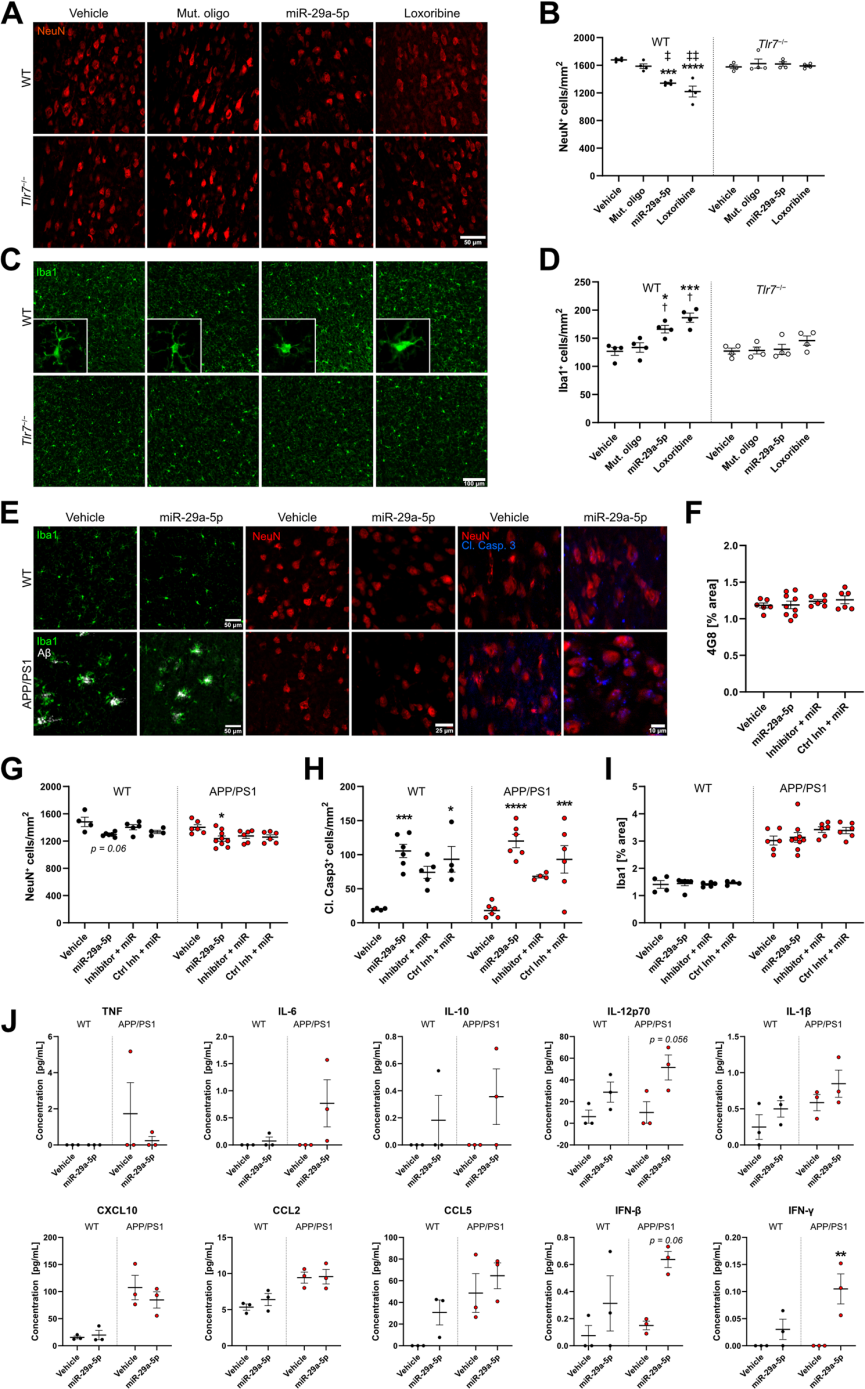
Intrathecal injection of miR-29a-5p into mice induces neuronal loss and microglial accumulation in the cerebral cortex.** A-D**) C57BL/6 (WT) and *Tlr7*^−/−^ mice were intrathecally injected with vehicle (water, 40 µL, *n* = 4 for each genotype), mut. oligo (10 µg, *n* = 4 for each genotype), miR-29a-5p (10 µg, *n* = 4 for each genotype), or loxoribine (136 µg, *n* = 4 for each genotype), and sacrificed after 3 d. **A**) Representative images of NeuN immunostaining of the cerebral cortex of WT and *Tlr7*^*−/−*^ mice. Scale bar represents 50 μm. **B**) Quantification of NeuN-positive neurons in the cerebral cortex. **C**) Representative images of Iba1 immunostaining in WT and *Tlr7*^*−/−*^ mice. Scale bar represents 100 μm. **D**) Quantification of Iba1-positive microglia in the cerebral cortex. **E-J**) WT and APP/PS1 mice given 3 monthly intrathecal injections of vehicle (water, 40 µL, *n* = 6), miR-29a-5p (10 µg, *n* = 9) with or without pre-treatment with LNA (125 pmol, *n* = 6) or control inhibitor (125 pmol, *n* = 6). **E**) Representative images of Iba1, 4G8, NeuN, and cleaved caspase-3 immunostaining in the cortex of vehicle- and miR-29a-5p-treated WT and APP/PS1 mice. Scale bar represents 50, 25, and 10 μm, respectively. **F**) Percent area of 4G8-positive Aβ plaques in the entire cortex of APP/PS1 mice. **G**) Quantification of NeuN-positive neurons in the cerebral cortex of WT and APP/PS1 mice. **H**) Quantification of caspase-3-positive cells in the cortex of WT and APP/PS1 mice. **I**) Percentage of Iba1-positive microglia coverage in the cortex of WT and APP/PS1 mice. **J**) Multiplex immunoassay analysing cyto- and chemokine expression in brain tissue from vehicle or miR-29a-5p conditions in WT and APP/PS1 mice (*n* = 3). Significance determined by a two-way ANOVA followed by Tukey’s multiple comparison test. *p < 0.05; **p < 0.01; ***p < 0.001; ****p < 0.0001 relative to vehicle control. ^†^*p* < 0.05; ^‡^*p* < 0.01; ^‡‡^ *p* < 0.0001 to *Tlr7*^*−/−*^ of the same condition. Error bars represent mean ± SEM. *n* = 3–9.

To investigate the effects of extracellularly delivered miR-29a-5p on the CNS in vivo, we intrathecally injected WT and *Tlr7*^−/−^ mice with synthetic miR-29a-5p. Additional mice were injected with the mut. oligo as a sequence specificity control, loxoribine as a positive control for TLR7 activation, or water as the vehicle sham control. After 3 d, mice were sacrificed, and cerebral cortices were analysed by immunohistochemistry. Immunostaining with NeuN antibody revealed neuronal loss in the cerebral cortex of WT mice injected with miR-29a-5p or loxoribine compared to both vehicle and mut. oligo controls. These neurotoxic effects induced by miR-29a-5p or loxoribine in WT mice were abolished in *Tlr7*^−/−^ mice (Fig. 4A, B). Dendritic and axonal structure, as assessed by immunostaining with MAP2 and neurofilament antibodies, respectively, were not altered by miR-29a-5p treatment of WT or *Tlr7*^−/−^ mice compared to control conditions (Fig. S4A-D). Quantification of Iba1-positive cells revealed increased numbers of microglia in the cerebral cortex of WT mice injected with miR-29a-5p or loxoribine compared to both vehicle and mut. oligo conditions. In contrast, microglial numbers did not differ in *Tlr7*^−/−^ mice in any condition (Fig. 4C, D), indicating a role for TLR7 in microglial accumulation in response to intrathecal miR-29a-5p. Besides microglia, GFAP-positive astrocytes also express TLR7 [51]. However, as GFAP is readily expressed by nearly all hippocampal but not cortical astrocytes [52], hippocampal GFAP-positive astrocytes were quantified in our experimental set-up. Neither miR-29a-5p nor loxoribine altered astrocyte coverage in WT or *Tlr7*^−/−^ mice (Fig. S4E, F).

To investigate the effect of miR-29a-5p as a signalling molecule in the context of AD in vivo, we injected both APP/PS1 and WT mice intrathecally with miR-29a-5p over an extended period of 12 weeks. To this end, 30 d old mice were treated with 3 monthly injections of miR-29a-5p and sacrificed 30 d after the final injection (Fig. S5A). RT-qPCR analysis of *miR-29a-5p* expression in the cerebral cortex of WT and APP/PS1 mice treated with miR-29a-5p mimic showed no changes in *miR-29a-5p* expression, likely due to degradation of the injected miRNA over the 30 d period since the final miRNA injection. At baseline, *miR-29a-5p* expression in APP/PS1 mice appeared lower than in WT, but this difference did not reach statistical significance (Fig. S5B). Next, Aβ plaque load in the cortex (Fig. 4E, F) and hippocampus (Fig. S5C) of APP/PS1 mice intrathecally injected with miR-29a-5p was quantified. No change in plaque load was observed in any tested group compared to control condition. Likewise, no substantial changes in the size distribution of plaques in the cortex or hippocampus were found in the different treatment conditions (Fig. S5D, E). Using a multiplex protein assay soluble and insoluble Aβ forms in the brains of miR-29a-5p-injected APP/PS1 mice were quantified. No significant changes in Aβ40 or Aβ42 expression were found (Fig. S5F). Aβ38, a vessel-associated Aβ subtype often present in cerebral amyloid angiopathy [53], was not detected in any condition (data not shown). This may be explained by the relatively young age of the injected mice (120 d), at which vascular Aβ expression levels are low [54].

As observed in the mouse model treated with a single miRNA injection (see Fig. 4A, B), neuronal numbers in the cerebral cortex of mice treated with multiple injections were reduced, with miR-29a-5p causing approximately a 12.5% loss of cortical neurons in APP/PS1 mice. In WT mice, we also observed such a trend (*p =* 0.06; Fig. 4G). No change in dendritic or axonal structure as measured by MAP2 and neurofilament expression, respectively, was observed in any condition (Fig. S5G-I). Pre-treatment with an LNA-based miR-29a-5p inhibitor prior to miR-29a-5p administration showed a slight trend towards preventing neuronal loss induced by miR-29a-5p alone in both APP/PS1 and WT mice, however, this effect was not statistically significant. Pre-treatment with a control inhibitor prior to miRNA injection resulted in a similar degree of neuronal injury as miR-29a-5p alone, though this effect did not reach statistical significance. Furthermore, cleaved caspase-3 expression was increased in both APP/PS1 and WT mice injected with miR-29a-5p (Fig. 4H). Pre-treatment with miR-29a-5p inhibitor reduced numbers of cleaved caspase-3-positive cells in both genotypes, although these effects did not reach statistical significance when compared to miR-29a-5p alone (WT, *p* = 0.56; APP/PS1, *p* = 0.09). In contrast to the mouse model of single miRNA injection described above (see Fig. 4D), repeated miR-29a-5p injections over 3 months did not change numbers of Iba1-positive microglia in the cerebral cortex (Fig. 4I) or hippocampus (Fig. S5K) of APP/PS1 or WT mice. Likewise, numbers of microglia expressing Dectin-1 were unchanged in APP/PS1 mice after 120 d of miRNA treatment (Fig. S5I, L). To further analyse the neuroinflammatory response of APP/PS1 and WT mice after multiple intrathecal miR-29a-5p injections over an extended time course, cytokine and chemokine expression in the respective cerebral cortex at time point 30 d after the last miRNA injection was determined by multiplex immunoassay (Fig. 4J, Table S2). MiR-29a-5p injection significantly increased IFN-γ expression in APP/PS1, but not in WT mice. IL-12p70 and IFN-β expression tended to increase in APP/PS1 mice treated with miR-29a-5p compared to vehicle (*p* = 0.056; *p* = 0.063, respectively). Expression levels of TNF, IL-6, IL-10, IL-1β, CXCL10, and CCL2 were unaltered by miRNA injections in both genotypes (Fig. 4J).

### Intrathecal miR-29a-5p downregulates the MAPK pathway in APP/PS1 and WT mice

**Fig. 5:**
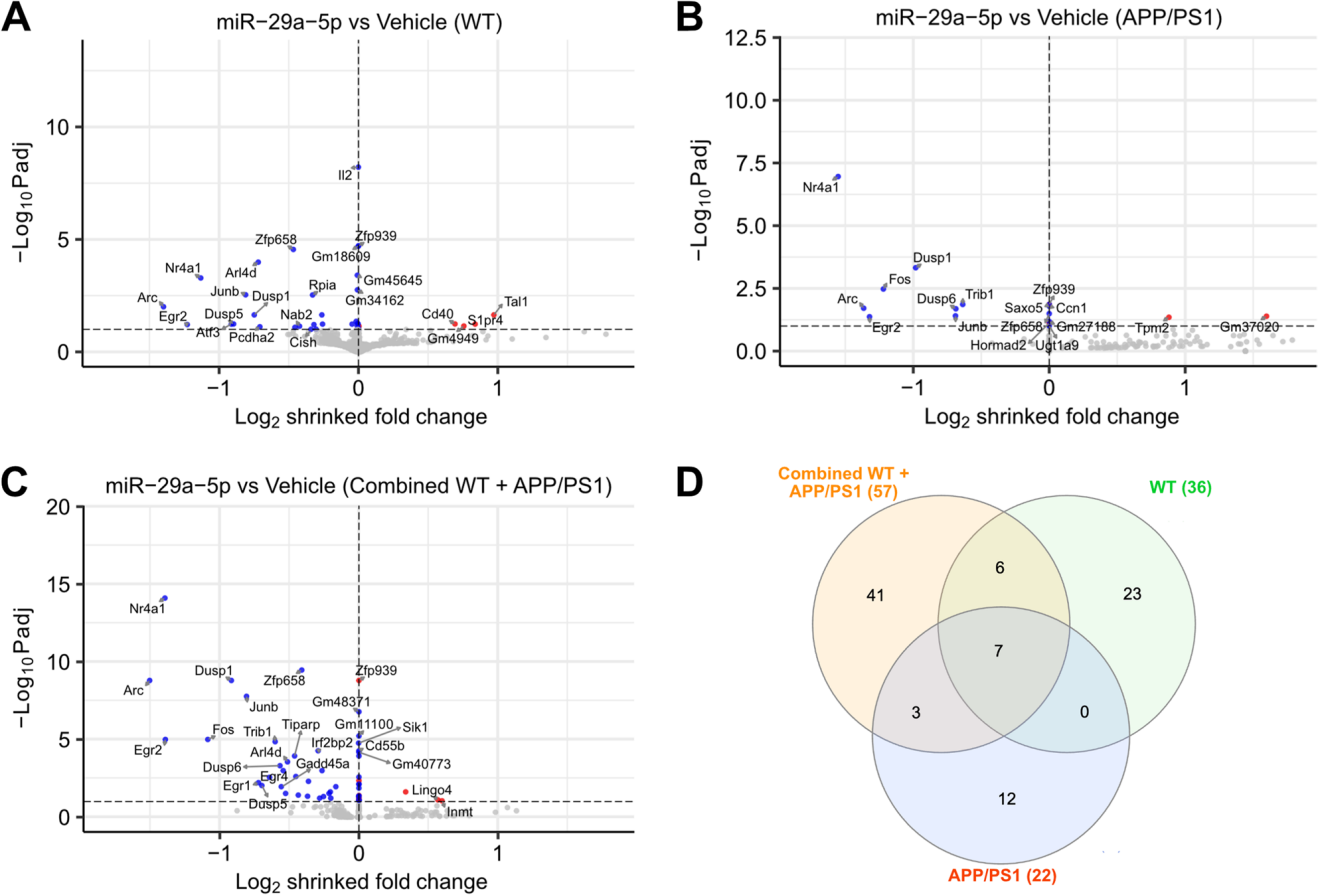
Inflammatory and transcriptional response of APP/PS1 and WT mice intrathecally injected with miR-29a-5p. Volcano plots of RNA-seq analysis showing differential gene expression from **A**) WT mice, **B**) APP/PS1 mice, or **C**) combined WT and APP/PS1 genotypes given three monthly injections of miR-29a-5p compared to vehicle. **D**) Venn diagram of RNA-seq analysis indicating the number of overlapping and divergent differentially expressed genes between WT, APP/PS1, and combined genotypes. *n* = 3 for all groups excluding *APP/PS1 inhibitor + miR-29a-5p* condition where *n* = 2.

Next, we sought to determine whether miR-29a-5p injections into mice result in transcriptomic changes in APP/PS1 and WT mice, over an extended period (time point 30 d after the third injection) (Fig. S7A). All differential gene expression was compared to the vehicle control. In both WT (Fig. 5A) and APP/PS1 (Fig. 5B) mice, miR-29a-5p treatment primarily downregulated MAPK pathway genes including *DUSP1*, *NR4A1*, and *Junb.* Similarly, miR-29a-5p in combination with LNA inhibitor or control inhibitor downregulated MAPK gene expression in both genotypes, except WT mice pre-treated with control inhibitor, where expression of only five genes was significantly affected (Table S2). The MAPK-inducible gene, *Arc*, which has been linked to synaptic plasticity, was also significantly downregulated in all treatment groups in both genotypes (Fig. 5A, B). Analysing genotype-specific gene differentiation linked to microglial function and AD after miR-29a-5p treatment, WT, but not APP/PS1, mice exhibited downregulation of *Cish*, a gene upregulated in AD patients and in response to Aβ stimulation in microglia [55], and upregulation of *TAL1*, a regulator of aging in microglia [56]. In APP/PS1, but not WT, mice, miR-29a-5p injections downregulated expression of *TRIB1*, a gene involved in TLR responses and M2 polarisation of macrophages [57, 58], and *CCN1*, which affects neuronal health in *App*^*NL−G−F*^ AD model mice [59] (Fig. 5A, B).

Given the consistent downregulation of MAPK pathway genes in both APP/PS1 and WT mice, we analysed both genotypes together to determine overall treatment effects of miR-29a-5p (Fig. 5C, D). All conditions resulted in downregulation in the aforementioned MAPK pathway genes (*Fos*,* Junb*,* NR4A1*,* DUSP1/5/6*), but additional MAPK genes such as *GADD45A* were also found to be downregulated. GO analysis of differential gene expression for combined genotypes injected with miR-29a-5p primarily showed downregulation of negative phosphorylation terms (Fig. S7B) and downregulation of genes related to the MSigDB pathway *TNF signalling via NFKB* (Fig. S7C). Finally, considering miRNA’s canonical function as regulators of post-transcriptional gene expression, we queried the databases miRDB and miRTarBase to search for mRNA targets of miR-29a-5p, which may confound our transcriptome data derived from mice injected with the miRNA described above. No theoretical or experimentally validated mRNA targets that are downregulated in APP/PS1 or WT mice treated with miR-29a-5p were detected (Fig. S7D).

## Discussion

AD is associated with miRNA dysregulation, but the functional consequences of this alteration are unknown. Recent studies have shown that *Tlr7 *expression is increased in both AD patients and multiple AD mouse models, suggesting a role for this receptor in AD pathology [60–62]. Given that numerous miRNAs activate TLR7, thereby driving inflammation, this interaction may provide a consequence of miRNA dysregulation in AD [10–12, 25, 27]. We therefore aimed to investigate the impact of miRNAs associated with AD and neuroinflammation as potential ligands for TLR7 in the CNS. We identified several miRNAs that directly activate mouse and human TLR7, but also its twin receptor in ssRNA sensing, human TLR8, and trigger microglial activation. Considering the central role of inflammation in AD pathology, we investigated if and how one of these specific miRNAs, miR-29a-5p, which is dysregulated in AD patients [63], triggers neuroinflammation, thereby potentially serving as an example for other miRNAs acting as signalling molecules in the CNS.

In our study, extracellularly applied miR-29a-5p caused microglial cyto- and chemokine release, improved phagocytosis of Aβ, and induced neuronal injury, dependent on TLR7. Notably, miR-29a-3p, the complementary variant of miR-29a-5p, has also been shown to activate TLR7 and induce cytokine release from immune cells [64], implying this is a common function of miR-29a. Our study implicates miR-29a-5p as an extracellularly active signalling molecule in CNS inflammation. Other research has shown anti-inflammatory effects of miR-29a-5p. For instance, in a model of brain injury, transfected miR-29a-5p reduced IL-1β production [65]. In a mouse model of depression, chronic miR-29a-5p overexpression shifted glia to a homeostatic state [66]. This data suggests that miR-29a-5p’s inflammatory effects are context-dependent and vary by disease model. Similar context-dependence has been found for other miRNAs, such as miR-223-3p, which we also validated as a TLR7 ligand. This miRNA reduces inflammation when either genetically deleted or when chronically overexpressed depending on the disease model [67, 68]. Importantly, these studies highlight the miRNAs’ canonical role in gene silencing rather than their function as extracellularly active signalling molecules. This may be the reason for the miRNA’s different effects on the inflammatory response. Our data indicate that extracellular miR-29a-5p can enter microglia to activate RNA-sensing TLRs located to the endosomal compartment, as previously described for other miRNAs [10, 29]. As such, for extracellular miRNAs to exert gene silencing functions, they would need to cross the endosomal lipid bilayer, which is a severe rate-limiting factor [69], and may also explain why we observe pro- rather than anti-inflammatory responses from miR-29a-5p acting as a signalling molecule for microglia. Even so, disease context should be carefully considered when analysing inflammation driven by miRNAs as this could substantially influence the potential therapeutic implications for miRNAs. Determining the potential of selected miRNAs as therapeutic targets in CNS diseases such as AD is in progress though challenges remain in delivery, specificity, and side effects [70]. Also, as miRNAs target multiple mRNAs in their canonical function, besides acting as TLR ligands, deciphering the downstream signalling consequences of miRNA therapeutics is highly complex. Thus, to target specific cell types and pathological conditions extensive preclinical validation is required. Neuronal injury is a hallmark of neuroinflammatory states, including AD. In this study, extracellular miR-29a-5p requires TLR7-expressing microglia to cause distinct inflammatory and neurotoxic effects in the murine brain. Our data suggest a role for yet unidentified neurotoxic molecules released from microglia upon miR-29a-5p stimulation. In principle, microglia can release neurotoxic factors including cytokines such as TNF, nitric oxide, among others, when activated by TLRs [49, 50]. We cannot rule out that some miR-29a-5p mimic remaining in the conditioned media used for incubation of neurons was, at least in part, responsible for the observed neurotoxicity. However, since we did not detect toxic effects from miR-29a-5p doses as high as 10 µg/ml in neuron cultures within similar time frames, such a scenario seems unlikely. Also, in an AD context, microglia might engulf dying neurons because of Aβ exposure. TLR7 is not only expressed in microglia but also in neurons and is involved in synapse physiology and cell death [21, 71]. Our previous data showed that TLR7-activating miRNAs such as *let-7b* and miR-100-5p, whose expression is upregulated in AD, induce TLR7-dependent neurotoxicity in a cell-autonomous fashion, without microglial involvement [10, 12]. In our current study, cell-autonomous toxic effects of miR-29a-5p on neurons were relatively delayed and mild compared to other TLR7-activating miRNAs [10, 12, 29], indicating that miRNA-TLR7 interactions in the brain have cell- and sequence-specific functional consequences. In our study, the mechanisms of CNS cell type-specific responses to miR-29a-5p treatment remain unresolved. How extracellular miRNAs act differently upon specific cell types, not only on microglia and neurons, but also on other CNS cells such as astrocytes and oligodendrocytes, and why the functional outcome of TLR7 activation in these different cell types differs, is unclear. Features such as high guanosine content or successive uridines present in the miRNA’s sequence may preferentially target the first and/or second binding site of TLR7, respectively [72, 73], thereby potentially modulating receptor activation and the downstream signalling cascades in a distinct cell type. Other mechanisms particularly relevant in vivo include cell-specific extracellular small RNA transfer which has been reported for miRNAs encapsulated within extracellular vesicles (EVs). Mast cells release miR-409-3p in EVs to promote microglial migration [74], while astrocyte-derived miR-873a-5p attenuates microglial inflammation [75], though in our experimental system miRNAs were not encapsulated in EVs. We have previously shown and now confirmed that CNS cells uptake non-EV-associated extracellular miRNAs, which co-localise to endosomes (and TLR7) [10], but how this applies to specific cell populations in heterogeneous organ systems like the CNS in vivo remains unresolved. Furthermore, in contrast to microglia cortical neurons are able to uptake uncomplexed miRNA, as reported previously [10, 12, 29]. We showed recently that dynamin-mediated endocytosis is involved in the uptake of certain neurodegenerative disease-associated naked miRNAs, such as miR-124-5p, by CNS neurons [29]. Future studies may resolve the sequence motifs and other miRNA features controlling extracellular miRNA uptake and trafficking mechanisms in different cell types.

So far, potential long-term and disease-specific effects of extracellular miRNAs in the CNS are poorly understood. We therefore modified our established mouse model of intrathecal injection, whereby 3 monthly injections of miR-29a-5p were given to WT and APP/PS1 mice. Unlike our acute single injection model, where intrathecal miR-29a-5p increased microglial numbers after 3 d, several miRNA injections given over an extended period did not change microglial marker expression or key pathologies of APP/PS1 mice, including expression of soluble and insoluble Aβ40/42, with Aβ38 being undetectable. As we did not evaluate the effects of our 3 d-single injection protocol in APP/PS1 mice, we cannot exclude the possibility of short-term effects from miR-29a-5p injection in this AD mouse model. Notably, the neurotoxic effects observed after a single injection of miR-29a-5p after 3 d persisted but were not cumulative with repeated injections over extended time. This suggests that miRNA-induced neuronal injury represents an acute rather than chronic effect. Also, the miRNA injection may have affected certain subpopulations of CNS neurons though whether injected miR-29a-5p or other miRNAs preferentially target specific neuronal types or brain regions remains unknown. Effects on microglial accumulation observed after 3 d miRNA treatment in the acute model were abolished in the experimental long-term setting, potentially due to degradation of the injected miR-29a-5p mimic over a 30 d-period after the final injection. Although the oligoribonucleotide we injected has been chemically stabilised by phosphorothioate bonds, which prevent RNA degradation, this protective effect may subside over time and thus, an effect on microglial numbers has not been detected after intrathecal miR-29a-5p injection in our long-term model. Although employing an established mouse model of intrathecal injection, it remains unclear, how the injected miRNA reaches the cerebral cortex. However, as intrathecal injection of mutant oligonucleotide did not have an effect on neuronal viability or microglia numbers, whereas miR-29a-5p and other sequence-specific miRNAs in previous studies did, it implies that in principle, miRNAs can reach the brain by intrathecal injection and induce sequence-specific effects [10, 12, 29]. Future studies are necessary to analyse time-dependent effects and dynamics of specific miRNA injection and especially, to determine therapeutic implications of such miRNAs acting as signalling molecules in the brain.

After 120 d miRNA treatment we observed an increase in the expression of inflammatory molecules, such as IFN-γ, and to a lesser extent of IFN-β and IL-12p70 in APP/PS1, but not in WT mice. IFN-γ, IFN-β, and IL-12 signalling have been shown to contribute to the pathology of APP/PS1 mice and may play a role in AD pathogenesis [76–78]. Altogether, our data demonstrates that extracellularly applied miR-29a-5p can induce long-term changes in the CNS inflammation pattern, though the cellular source responsible for these changes remains undetermined. As such, the mechanistic link between intrathecal miR-29a-5p application and the increase in the expression of inflammatory molecules also remains unresolved. We only detected alterations of the inflammatory pattern in APP/PS1 mice, but not in WT mice, suggesting that the cellular environment alters the inflammatory response to miR-29a-5p treatment. Such observations have been reported in other studies using LPS, e.g [79]. Future studies will be required to dissect the effects of miRNAs on different CNS cell types and genotypes. For example, employing a conditional *Tlr7*^−/−^ knockout mouse model and AD models lacking TLR7 expression may validate the role of microglia in miR-29a-5p-induced neuronal injury and modulation of the inflammatory response, particularly in an AD context.

So far, the endogenous miRNA concentrations in the brain, which would be sufficient to activate TLR7, are unknown. We have previously shown that 2 million neurons release approx. 22.89 nM of miRNA (6.9 million copies/neuron) [10], and in this study found that miR-29a-5p causes microglial TNF release starting at doses from 0.1 µg/mL miRNA (approx. 11.43 nM; *p* = 0.001, unpaired *t-*test, miRNA compared to unstimulated condition; see Fig. 2A). Given this, it is plausible that miRNA amounts released from dying neurons — and potentially from other CNS cell types — are sufficient to activate TLR7, particularly in pathological conditions when neurons degenerate. Notably, miR-29a-5p is expressed at higher levels in microglia compared to neurons [80, 81]. Still, in our study, we cannot rule out that supraphysiological doses of miR-29a-5p causing the observed effects in vitro and in vivo were used. Furthermore, whether the effects from miR-29a-5p application on CNS cells were direct or secondary, involving yet unidentified signalling pathways, remains unresolved. An increase in miR-29a-5p expression in WT or APP/PS1 mice injected with miR-29a-5p mimic was not detected by qPCR, though this might be, at least in part, due to degradation of the administered miRNA. In addition, the respective cellular response to extracellular miRNA may be altered upon repeated doses. Future research may determine the concentrations of both endogenous and applied miRNAs required to activate TLR7 in the brain, both in homeostasis and disease.

Interrogating transcriptional changes revealed intrathecal miR-29a-5p caused enduring downregulation in MAPK pathway-related genes such as *NR4A1*, *DUSP*, *Junb*, and *Fos*, in both WT and APP/PS1 mice. MAPK is a fundamental pathway involved in many processes including apoptosis, proliferation, and inflammation. Consequently, aberrant regulation of the pathway has been implicated in many diseases including AD, and further neurodegenerative diseases such as amyotrophic lateral sclerosis [82]. As such, MAPK inhibitors are being investigated as clinical compounds for AD [83]. Notably, *DUSP* genes, including *DUSP1* and *6*, which are downregulated in AD patients and modulate the amyloidogenic process [84–86], are critical inhibitors of the MAPK pathway activity and were downregulated by miR-29a-5p administration. Importantly, none of the differentially expressed genes from miR-29a-5p treatment are known or predicted mRNA targets of miR-29a-5p, suggesting that MAPK pathway deregulation is driven by direct miRNA–TLR interaction, although this cannot be definitively confirmed here. Still, the fact that the MAPK pathway modulates microglia-driven inflammation, is activated by, and helps to regulate TLR signalling [87–89], does suggest miR-29a-5p acting as a signalling molecule alters TLR signalling. A TLR-mediated mechanism is further supported by downregulation of specific genes like *Arc*, which is involved in neuroinflammation [90] and is downregulated upon TLR7/8 activation [91], and *NR4A1*, which prevents overactivation of microglia from TLR signalling [92, 93]. Of note, many of these genes were also downregulated when both miR-29a-5p and its LNA inhibitor were administered. This may be due to the fact that the inhibitor was pre-administered 16 h before miR-29a-5p and thereby, could be depleted by endogenous miRNA, resulting in reduced inhibition of the subsequent injection of synthetic miR-29a-5p. In line with this, there was no significant effect of the LNA-based miRNA inhibitor on neurotoxicity induced by the injected miRNA in both WT and APP/PS1 mice. Although single genes were differentially expressed in WT and APP/PS1 mice in response to miRNA treatment and even with the differing cellular environments of both genotypes, miR-29a-5p broadly downregulated the MAPK pathway, indicating a common mechanism of action of miR-29a-5p, when acting as a signalling molecule in both genotypes.

MiR-29a-5p is associated with a wide range of diseases from colorectal and liver cancer to glioma, exemplifying the broad consequences of miRNA dysregulation [94–96]. In AD patients, the expression of miR-29a is frequently dysregulated and has been proposed as a possible biomarker for disease progression [63, 97–100]. MiR-29a-3p is downregulated in the brain of AD patients with high levels of BACE1 [97] and in the serum of probable AD patients [99], but conversely, increased in the plasma [98] and CSF of AD patients [101]. MiR-29a-5p expression is negatively correlated with cortical thickness in AD patients [63]. Furthermore, the expression of miR-29a-5p in neuronal and microglial EVs is reduced in AD patients [63]. This is crucial when considering miR-29a-5p as an extracellularly active molecule, as miRNAs in EVs play a central role in mediating immune responses [102]. Given both miR-29a-5p and miR-29a-3p are downregulated in the brain, and they both activate TLR7 causing cytokine release, reduced expression of miR-29a in AD patient brains could be a protective mechanism to limit inflammation via TLR7.

## Conclusion

This study identifies certain miRNAs dysregulated in AD patients and neuroinflammation as TLR7 ligands capable of activating microglia, thereby shaping the neuroinflammatory response. Extracellularly delivered AD-associated miR-29a-5p induces lasting alterations of the expression of certain cytokines and MAPK pathway elements in the murine brain. Our findings indicate that extracellular miRNA can have prolonged effects in an AD context, supporting the miRNAs’ potential as candidate molecules relevant for both disease pathogenesis and novel therapeutic approaches. Future research exploring the pharmacokinetics/dynamics, trafficking, and cell-specific effects of extracellular miRNAs in vivo will be vital to understand the outcome of miRNA dysregulation in neuroinflammatory states and for the miRNAs’ progress as disease-modifying tools.

## Supplementary Information

Supplementary Material 1. Figure S1. LyoVec transfection increases microglial TNF response to miR-29a-5p, and miR-29a-5p co-localises to endosomes in microglia. A) C57BL/6 (WT) microglia were exposed to free or LyoVec-transfected miR-29a-5p and mutant oligonucleotide (10 μg/mL) for 24 h. TNF concentration in the supernatant was subsequently assessed by ELISA. LPS (100 ng/mL) served as a positive control (*n* = 3). B) WT microglia were incubated with pHrodo Red (20 μg/mL) and Alexa-488-conjugated miR-29a-5p (10 μg/mL) for 4 h with line profiles depicting fluorescence intensity in marked region of interest (white line). Figure S2. Extracellular miR-29a-5p induces transcriptional TLR pathway changes in microglia in vitro. A) WT and *Tlr7*^−/−^ microglia were exposed to LPS (100 ng/mL) for 24 h. B) WT microglia were exposed to LPS (100 ng/mL) or miR-29a-5p (10 μg/mL) for 6 h. C) WT neurons were exposed to miR-29a-5p (10 μg/mL) for 48 h. D) miR-29a-5p expression was assessed in WT microglia exposed to miR-29a-5p (10 μg/mL), Aβ (10 μM), or loxoribine (1 mM) for 24 h. For all experiments, RNA was extracted from cells for analysis by RT-qPCR. Significance tested by multiple unpaired t-tests by two-stage step-up method A-C) or one-way ANOVA followed by Sidak’s multiple comparison test D). **p < 0.01 to unstimulated, ^†^*p* < 0.05 to gene of interest exposed to different conditions. Dashed line represents unstimulated condition. Error bars represent mean ± SEM. *n* = 3. Figure S3. Effect of extracellular miR-29a-5p on cortical neurons in vitro. A) Representative images of NeuN and Iba1 immunostaining after 5 d in co-cultures of WT neurons and WT microglia exposed to mut. oligo, miR-29a-5p (10 μg/mL), or loxoribine (1 mM). Scale bar represents 50 μM. B, C) Cultures of WT microglia were stimulated with mut. oligo, miR-29a-5p (10 μg/mL), or loxoribine (1 mM) for 24 h to create conditioned media. Cultures of enriched cortical neurons were exposed to this microglia-conditioned media for 5 d. B) Representative images of NeuN and DAPI and C) quantification of immunostaining in cultures of enriched cortical neurons after 5 d of exposure to microglial conditioned media. Scale bar represents 50 μM. D) Representative images of NeuN immunostaining, DAPI, and TUNEL staining in enriched WT neurons after stimulation with indicated agent for 5 d. Scale bar represents 50 μm. E) Enriched cortical neurons were treated with miR-29a-5p (10 μg/mL) for 5 or 8 d, immunostained with NeuN antibody, and stained with TUNEL assay and DAPI. Ratio of TUNEL- to DAPI-positive cells was assessed. Mut. oligo (10 μg/mL) and loxoribine (1 mM) served as controls. F) Quantification of miR-29a-5p expression in WT neurons exposed to miR-29a-5p mimic (10 μg/mL), Aβ (10 μM), or loxoribine (1 mM) for 24 h, by RT-qPCR. Dashed line represents unstimulated condition. Significance tested by one-way ANOVA followed by Sidak’s multiple comparison test. *p < 0.05; **p < 0.01; ***p < 0.001; ****p < 0.0001 to unstimulated or ‡*p* < 0.01 to mut. oligo condition. *n* = 3-5. Figure S4. Intrathecal injection of miR-29a-5p does not affect cortical dendrite and axon structure, or hippocampal astrocyte numbers. A-F) WT and Tlr7^−/−^ mice were injected with vehicle (water, 40 μL), mut. oligo (10 μg), miR-29a-5p (10 μg), or loxoribine (136 μg) and sacrificed after 3 d. A) Representative images of MAP2 immunostaining in the retrosplenial cortex. B) Quantification of cortical MAP2-positive dendrites. C) Representative images of neurofilament immunostaining in the retrosplenial cortex. D) Quantification of neurofilament-positive axons in the cortex E) Representative images of hippocampal GFAP immunostaining. F) Quantification of GFAP-positive astrocytes in the hippocampus. Scale bars represent 100 μm for MAP2 and NF or 500 μm for GFAP. Error bars represent mean ± SEM. *n* = 4. Figure S5. miR-29a-5p administration has no effect on plaque accumulation or neurite structure in WT and APP/PS1 mice. A) Timeline of repeated intrathecal injections into WT and APP/PS1 mice. B) Quantification of miR-29a-5p expression by RT-qPCR in WT and APP/PS1 mice treated with vehicle or miR-29a-5p mimic, as described in Fig. 4 E-J). C) 4G8-positive Aβ plaque area in the hippocampus of APP/PS1 mice. D) Distribution of 4G8-positive Aβ plaque size in the cortex or E) hippocampus of APP/PS1 mice. F) Multiplex analysis of soluble and insoluble Aβ40 and Aβ42 in cortical tissue of WT and APP/PS1 mice treated with vehicle or miR-29a-5p. G) Representative images of MAP2 and neurofilament immunostaining of the cerebral cortex of WT and APP/PS1 mice. Scale bars represent 50 μm. H) Quantification of MAP2-positive dendrites in the cortex of WT and APP/PS1 mice. I) Quantification of neurofilament-positive axons in the cortex of WT and APP/PS1 mice. J) Representative images of Iba1 and Dectin-1 immunostaining of the cortex of APP/PS1 mice. Scale bar represents 50 μm. K) Quantification of Iba1-positive microglia in the hippocampus of WT and APP/PS1 mice. L) Quantification of Dectin-1-positive microglia in the cerebral cortex of APP/PS1 mice. Error bars represent mean ± SEM. *n* = 4-9. Figure S6. Clustering and GO analysis of APP/PS1 and WT mice. A) Final clustering heatmap of all analysed mice intrathecally injected with 3 monthly doses of miR-29a-5p. B) GO analysis of combined genotypes after repeated miR-29a-5p administration. C) MsigDB analysis of differentially expressed genes from combined genotypes for miR-29a-5p administration. D) Theoretical and experimentally validated mRNA targets of miR-29a-5p that are differentially expressed in RNA-seq analysis. *n* = 3 for all groups excluding APP/PS1 inhibitor + miR-29a-5p condition where *n* = 2.

Supplementary Material 2. Table S1. List of reagents (antibodies, primers, cell lines, chemicals, and proteins) used in experimental procedures.

Supplementary Material 3. Table S2. Excel file containing complete data for differentially expressed genes in WT and APP/PS1 mice, mouse characteristics, mRNA gene targets of miR-29a-5p, and cyto-/chemokine analysis.

## Data Availability

The datasets used in the current study are available from the corresponding author on reasonable request.

## References

[1] Peng Y, Croce CM. The role of MicroRNAs in human cancer. Sig Transduct Target Ther. 2016;1:1–9. 10.1038/sigtrans.2015.4 PMC5661652 29263891

[2] Peters LJF, Biessen EAL, Hohl M, Weber C, van der Vorst EPC, Santovito D. Small things matter: relevance of MicroRNAs in cardiovascular disease. Front Physiol. 2020;11:793. 32733281 10.3389/fphys.2020.00793 PMC7358539

[3] Pauley KM, Cha S, Chan EKL. MicroRNA in autoimmunity and autoimmune diseases. J Autoimmun. 2009;32:189–94. 19303254 10.1016/j.jaut.2009.02.012 PMC2717629

[4] Kamal MA, Mushtaq G, Greig NH. Current update on synopsis of MiRNA dysregulation in neurological disorders. CNS Neurol Disord Drug Targets. 2015;14:492–501. 25714967 10.2174/1871527314666150225143637 PMC5878050

[5] Condrat CE, Thompson DC, Barbu MG, Bugnar OL, Boboc A, Cretoiu D, Suciu N, Cretoiu SM, Voinea SC. MiRNAs as biomarkers in disease: latest findings regarding their role in diagnosis and prognosis. Cells. 2020;9:276. 31979244 10.3390/cells9020276 PMC7072450

[6] Chia SY, Vipin A, Ng KP, Tu H, Bommakanti A, Wang BZ, Tan YJ, Zailan FZ, Ng ASL, Ling S-C, Okamura K, Tan E-K, Kandiah N, Zeng L. Upregulated blood miR-150-5p in alzheimer’s disease dementia is associated with Cognition, cerebrospinal fluid Amyloid-β, and cerebral atrophy. J Alzheimers Dis. 2022;88:1567–84. 35811521 10.3233/JAD-220116

[7] Tan YJ, Wong BYX, Vaidyanathan R, Sreejith S, Chia SY, Kandiah N, Ng ASL, Zeng L. Altered cerebrospinal fluid Exosomal MicroRNA levels in Young-Onset alzheimer’s disease and frontotemporal dementia. J Alzheimers Dis Rep. 2021;5:805–13. 34870106 10.3233/ADR-210311 PMC8609483

[8] Herrera-Espejo S, Santos-Zorrozua B, Álvarez-González P, Lopez-Lopez E, Garcia-Orad Á. A systematic review of MicroRNA expression as biomarker of Late-Onset alzheimer’s disease. Mol Neurobiol. 2019;56:8376–91. 31240600 10.1007/s12035-019-01676-9

[9] Kanach C, Blusztajn JK, Fischer A, Delalle I. MicroRNAs as candidate biomarkers for alzheimer’s disease. Noncoding RNA. 2021;7:8. 33535543 10.3390/ncrna7010008 PMC7930943

[10] Wallach T, Mossmann ZJ, Szczepek M, Wetzel M, Machado R, Raden M, Miladi M, Kleinau G, Krüger C, Dembny P, Adler D, Zhai Y, Kumbol V, Dzaye O, Schüler J, Futschik M, Backofen R, Scheerer P, Lehnardt S. MicroRNA-100-5p and microRNA-298-5p released from apoptotic cortical neurons are endogenous Toll-like receptor 7/8 ligands that contribute to neurodegeneration. Mol Neurodegener. 2021;16:80. 34838071 10.1186/s13024-021-00498-5 PMC8626928

[11] McGurran H, Kumbol V, Krüger C, Wallach T, Lehnardt S. miR-154-5p is a novel endogenous ligand for TLR7 inducing microglial activation and neuronal injury. Cells. 2024;13:407. 38474371 10.3390/cells13050407 PMC10930870

[12] Lehmann SM, Krüger C, Park B, Derkow K, Rosenberger K, Baumgart J, Trimbuch T, Eom G, Hinz M, Kaul D, Habbel P, Kälin R, Franzoni E, Rybak A, Nguyen D, Veh R, Ninnemann O, Peters O, Nitsch R, Heppner FL, Golenbock D, Schott E, Ploegh HL, Wulczyn FG, Lehnardt S. An unconventional role for mirna: let-7 activates Toll-like receptor 7 and causes neurodegeneration. Nat Neurosci. 2012;15:827–35. 22610069 10.1038/nn.3113

[13] He S, Chu J, Wu L-C, Mao H, Peng Y, Alvarez-Breckenridge CA, Hughes T, Wei M, Zhang J, Yuan S, Sandhu S, Vasu S, Benson DM, Hofmeister C, He C, Ghoshal X, Devine K, Caligiuri SM, Yu MA J. MicroRNAs activate natural killer cells through Toll-like receptor signaling. Blood. 2013;121:4663–71. 23580661 10.1182/blood-2012-07-441360 PMC3674667

[14] Kumar V. Toll-like receptors in the pathogenesis of neuroinflammation. J Neuroimmunol. 2019;332:16–30. 30928868 10.1016/j.jneuroim.2019.03.012

[15] Shao Y, Saredy J, Yang WY, Sun Y, Lu Y, Saaoud F, Drummer C, Johnson C, Xu K, Jiang X, Wang H, Yang X. Vascular endothelial cells and innate immunity. Arterioscler Thromb Vasc Biol. 2020;40:e138–52. 32459541 10.1161/ATVBAHA.120.314330 PMC7263359

[16] Liu S, Liu Y, Hao W, Wolf L, Kiliaan AJ, Penke B, Rübe CE, Walter J, Heneka MT, Hartmann T, Menger MD, Fassbender K. TLR2 is a primary receptor for alzheimer’s amyloid β peptide to trigger neuroinflammatory activation. J Immunol. 2012;188:1098–107. 22198949 10.4049/jimmunol.1101121

[17] Miron J, Picard C, Frappier J, Dea D, Théroux L, Poirier J. TLR4 gene expression and Pro-Inflammatory cytokines in alzheimer’s disease and in response to hippocampal deafferentation in rodents. J Alzheimers Dis. 2018;63:1547–56. 29782315 10.3233/JAD-171160

[18] Zhou J, Yu W, Zhang M, Tian X, Li Y, Lü Y. Imbalance of microglial TLR4/TREM2 in LPS-Treated APP/PS1 Transgenic mice: A potential link between alzheimer’s disease and systemic inflammation. Neurochem Res. 2019;44:1138–51. 30756214 10.1007/s11064-019-02748-x

[19] Delgado MA, Elmaoued RA, Davis AS, Kyei G, Deretic V. Toll-like receptors control autophagy. EMBO J. 2008;27:1110–21. 18337753 10.1038/emboj.2008.31 PMC2323261

[20] Hung Y-F, Chen C-Y, Li W-C, Wang T-F, Hsueh Y-P. Tlr7 deletion alters expression profiles of genes related to neural function and regulates mouse behaviors and contextual memory. Brain Behav Immun. 2018;72:101–13. 29885943 10.1016/j.bbi.2018.06.006

[21] Mukherjee P, Winkler CW, Taylor KG, Woods TA, Nair V, Khan BA, Peterson KE. SARM1, not MyD88, mediates TLR7/TLR9-induced apoptosis in neurons. J Immunol. 2015;195:4913–21. 26423149 10.4049/jimmunol.1500953 PMC4769638

[22] Squillace S, Salvemini D. Toll-like receptor-mediated neuroinflammation: relevance for cognitive dysfunctions. Trends Pharmacol Sci. 2022;43:726–39. 35753845 10.1016/j.tips.2022.05.004 PMC9378500

[23] Heppner FL, Ransohoff RM, Becher B. Immune attack: the role of inflammation in alzheimer disease. Nat Rev Neurosci. 2015;16:358–72. 25991443 10.1038/nrn3880

[24] Melchiorri D, Merlo S, Micallef B, Borg J-J, Dráfi F. Alzheimer’s disease and neuroinflammation: will new drugs in clinical trials pave the way to a multi-target therapy? Front Pharmacol. 2023;14:1196413. 37332353 10.3389/fphar.2023.1196413 PMC10272781

[25] Gambuzza ME, Sofo V, Salmeri FM, Soraci L, Marino S, Bramanti P. Toll-like receptors in alzheimer’s disease: a therapeutic perspective. CNS Neurol Disord Drug Targets. 2014;13:1542–58. 25106635 10.2174/1871527313666140806124850

[26] Leng F, Edison P. Neuroinflammation and microglial activation in alzheimer disease: where do we go from here? Nat Rev Neurol. 2021;17:157–72. 33318676 10.1038/s41582-020-00435-y

[27] Raden M, Wallach T, Miladi M, Zhai Y, Krüger C, Mossmann ZJ, Dembny P, Backofen R, Lehnardt S. Structure-aware machine learning identifies MicroRNAs operating as Toll-like receptor 7/8 ligands. RNA Biol. 2021;18:268–77. 34241565 10.1080/15476286.2021.1940697 PMC8677043

[28] Capaccioli S, Dipasquale G, Mini E, Mazzei T, Quattrone A. Cationic lipids improve antisense oligonucleotide uptake and prevent degradation in cultured cells and in human serum. Biochem Biophys Res Commun. 1993;197:818–25. 8267621 10.1006/bbrc.1993.2552

[29] Kumbol V, Ivanov A, McGurran H, Schüler J, Zhai Y, Ludwik K, Hinkelmann L, Brehm M, Krüger C, Küchler J, Wallach T, Höltje M, Beule D, Stachelscheid H, Lehnardt S. Neurodegenerative disease-associated MicroRNAs acting as signaling molecules modulate CNS neuron structure and viability. Cell Commun Signal. 2025;23:196. 40275260 10.1186/s12964-025-02199-8 PMC12020182

[30] Stine WB, Dahlgren KN, Krafft GA, LaDu MJ. In vitro characterization of conditions for Amyloid-β peptide oligomerization and Fibrillogenesis *. J Biol Chem. 2003;278:11612–22. 12499373 10.1074/jbc.M210207200

[31] Stine WB, Jungbauer L, Yu C, LaDu MJ. Preparing synthetic Aβ in different aggregation States. Methods Mol Biol. 2011;670:13–32. 20967580 10.1007/978-1-60761-744-0_2 PMC3752843

[32] Choi Y, Joh Y, Ryu JS, Kim K, Seo D, Kim S. Endogenous Aβ peptide promote Aβ oligomerization tendency of spiked synthetic Aβ in alzheimer’s disease plasma. Mol Cell Neurosci. 2021;111:103588. 33422673 10.1016/j.mcn.2021.103588

[33] Hochmair J, Exner C, Franck M, Dominguez-Baquero A, Diez L, Brognaro H, Kraushar ML, Mielke T, Radbruch H, Kaniyappan S, Falke S, Mandelkow E, Betzel C, Wegmann S. Molecular crowding and RNA synergize to promote phase separation, microtubule interaction, and seeding of Tau condensates. EMBO J. 2022;41:e108882. 35298090 10.15252/embj.2021108882 PMC9156969

[34] Barghorn S, Biernat J, Mandelkow E. Purification of Recombinant Tau protein and Preparation of Alzheimer-paired helical filaments in vitro. Methods Mol Biol. 2005;299:35–51. 15980594 10.1385/1-59259-874-9:035

[35] Lian H, Roy E, Zheng H. Microglial Phagocytosis Assay. Bio-protocol. 2016;6:e1988. 10.21769/BioProtoc.1988 PMC5669280 29104891

[36] Schindelin J, Arganda-Carreras I, Frise E, Kaynig V, Longair M, Pietzsch T, Preibisch S, Rueden C, Saalfeld S, Schmid B, Tinevez J-Y, White DJ, Hartenstein V, Eliceiri K, Tomancak P, Cardona A. Fiji: an open-source platform for biological-image analysis. Nat Methods. 2012;9:676–82. 22743772 10.1038/nmeth.2019 PMC3855844

[37] Hoffmann O, Braun JS, Becker D, Halle A, Freyer D, Dagand E, Lehnardt S, Weber JR. TLR2 mediates neuroinflammation and neuronal damage. J Immunol. 2007;178:6476–81. 17475877 10.4049/jimmunol.178.10.6476

[38] Ewels P, Magnusson M, Lundin S, Käller M. MultiQC: summarize analysis results for multiple tools and samples in a single report. Bioinformatics. 2016;32:3047–8. 27312411 10.1093/bioinformatics/btw354 PMC5039924

[39] Dobin A, Davis CA, Schlesinger F, Drenkow J, Zaleski C, Jha S, Batut P, Chaisson M, Gingeras TR. STAR: ultrafast universal RNA-seq aligner. Bioinformatics. 2013;29:15–21. 23104886 10.1093/bioinformatics/bts635 PMC3530905

[40] Li H, Handsaker B, Wysoker A, Fennell T, Ruan J, Homer N, Marth G, Abecasis G, Durbin R, 1000 Genome Project Data Processing Subgroup. The sequence Alignment/Map format and samtools. Bioinformatics. 2009;25:2078–9. 19505943 10.1093/bioinformatics/btp352 PMC2723002

[41] Pertea M, Pertea GM, Antonescu CM, Chang T-C, Mendell JT, Salzberg SL. StringTie enables improved reconstruction of a transcriptome from RNA-seq reads. Nat Biotechnol. 2015;33:290–5. 25690850 10.1038/nbt.3122 PMC4643835

[42] Zhu A, Ibrahim JG, Love MI. Heavy-tailed prior distributions for sequence count data: removing the noise and preserving large differences. Bioinformatics. 2019;35:2084–92. 30395178 10.1093/bioinformatics/bty895 PMC6581436

[43] Wallach T, Wetzel M, Dembny P, Staszewski O, Krüger C, Buonfiglioli A, Prinz M, Lehnardt S. Identification of CNS Injury-Related MicroRNAs as novel Toll-Like receptor 7/8 signaling activators by small RNA sequencing. Cells. 2020;9:186. 31940779 10.3390/cells9010186 PMC7017345

[44] Liu J, Xu C, Hsu L-C, Luo Y, Xiang R, Chuang T-H. A five-amino-acid motif in the undefined region of the TLR8 ectodomain is required for species-specific ligand recognition. Mol Immunol. 2010;47:1083–90. 20004021 10.1016/j.molimm.2009.11.003 PMC2815190

[45] Fiebich BL, Batista CRA, Saliba SW, Yousif NM, de Oliveira ACP. Role of microglia TLRs in neurodegeneration. Front Cell Neurosci. 2018;12:329. 30333729 10.3389/fncel.2018.00329 PMC6176466

[46] Heil F, Ahmad-Nejad P, Hemmi H, Hochrein H, Ampenberger F, Gellert T, Dietrich H, Lipford G, Takeda K, Akira S, Wagner H, Bauer S. The Toll-like receptor 7 (TLR7)-specific stimulus loxoribine uncovers a strong relationship within the TLR7, 8 and 9 subfamily. Eur J Immunol. 2003;33:2987–97. 14579267 10.1002/eji.200324238

[47] Byrnes AE, Dominguez SL, Yen C-W, Laufer BI, Foreman O, Reichelt M, Lin H, Sagolla M, Hötzel K, Ngu H, Soendergaard C, Estevez A, Lin H-C, Goyon A, Bian J, Lin J, Hinz FI, Friedman BA, Easton A, Hoogenraad CC. Lipid nanoparticle delivery limits antisense oligonucleotide activity and cellular distribution in the brain after intracerebroventricular injection. Mol Ther Nucleic Acids. 2023;32:773–93. 37346977 10.1016/j.omtn.2023.05.005 PMC10280097

[48] McNab F, Mayer-Barber K, Sher A, Wack A, O’Garra A. Type I interferons in infectious disease. Nat Rev Immunol. 2015;15:87–103. 25614319 10.1038/nri3787 PMC7162685

[49] Lehnardt S, Schott E, Trimbuch T, Laubisch D, Krueger C, Wulczyn G, Nitsch R, Weber JR. A vicious cycle involving release of heat shock protein 60 from injured cells and activation of Toll-Like receptor 4 mediates neurodegeneration in the CNS. J Neurosci. 2008;28:2320–31. 18322079 10.1523/JNEUROSCI.4760-07.2008 PMC6671170

[50] Derkow K, Krüger C, Dembny P, Lehnardt S. Microglia induce neurotoxic IL-17 + γδ T cells dependent on TLR2, TLR4, and TLR9 activation. PLoS ONE. 2015;10:e0135898. 26288016 10.1371/journal.pone.0135898 PMC4545749

[51] Butchi NB, Du M, Peterson KE. Interactions between TLR7 and TLR9 agonists and receptors regulate innate immune responses by astrocytes and microglia. Glia. 2010;58:650–64. 19998480 10.1002/glia.20952 PMC3767435

[52] Zhang Z, Ma Z, Zou W, Guo H, Liu M, Ma Y, Zhang L. The Appropriate Marker for Astrocytes: Comparing the Distribution and Expression of Three Astrocytic Markers in Different Mouse Cerebral Regions. Biomed Res Int. 2019; 2019:9605265. 10.1155/2019/9605265 PMC6613026 31341912

[53] Reinert J, Martens H, Huettenrauch M, Kolbow T, Lannfelt L, Ingelsson M, Paetau A, Verkkoniemi-Ahola A, Bayer TA, Wirths O. Aβ38 in the brains of patients with sporadic and Familial alzheimer’s disease and Transgenic mouse models. J Alzheimer’s Disease. 2014;39:871–81. 24305500 10.3233/JAD-131373

[54] Klohs J, Rudin M, Shimshek DR, Beckmann N. Imaging of cerebrovascular pathology in animal models of alzheimer’s disease. Front Aging Neurosci. 2014;6:32. 10.3389/fnagi.2014.00032 PMC3952109 24659966

[55] Walker DG, Whetzel AM, Lue L-F. Expression of suppressor of cytokine signaling genes in human elderly and alzheimer’s disease brains and human microglia. Neuroscience. 2015;302:121–37. 25286386 10.1016/j.neuroscience.2014.09.052 PMC4385752

[56] Wehrspaun CC, Haerty W, Ponting CP. Microglia recapitulate a hematopoietic master regulator network in the aging human frontal cortex. Neurobiol Aging. 2015;36:e24439–244320. 10.1016/j.neurobiolaging.2015.04.008 PMC4503803 26002684

[57] Danger R, Feseha Y, Brouard S. The pseudokinase TRIB1 in immune cells and associated disorders. Cancers (Basel). 2022;14:1011. 35205759 10.3390/cancers14041011 PMC8869936

[58] Yamamoto M, Uematsu S, Okamoto T, Matsuura Y, Sato S, Kumar H, Satoh T, Saitoh T, Takeda K, Ishii KJ, Takeuchi O, Kawai T, Akira S. Enhanced TLR-mediated NF-IL6–dependent gene expression by Trib1 deficiency. J Exp Med. 2007;204:2233–9. 17724128 10.1084/jem.20070183 PMC2118688

[59] Hirabayashi S, Uyeda A, Manabe I, Yonezu Y, Saito T, Saido TC, Misawa H, Ogasawara Y, Kinoshita K, Muramatsu R. CCN1 derived from vascular endothelial cells impairs cognitive function in alzheimer’s disease model mice. J Pharmacol Sci. 2025;157:146–55. 39929589 10.1016/j.jphs.2025.01.004

[60] López-González I, Schlüter A, Aso E, Garcia-Esparcia P, Ansoleaga B, LLorens F, Carmona M, Moreno J, Fuso A, Portero-Otin M, Pamplona R, Pujol A, Ferrer I. Neuroinflammatory signals in alzheimer disease and APP/PS1 Transgenic mice: correlations with Plaques, Tangles, and oligomeric species. J Neuropathol Exp Neurol. 2015;74:319–44. 25756590 10.1097/NEN.0000000000000176

[61] Yu Liu H, Fen Hung Y, Ru Lin H, Li Yen T, Hsueh YP. Tlr7 Deletion Selectively Ameliorates Spatial Learning but does not Influence beta Deposition and Inflammatory Response in an Alzheimers Disease Mouse Model. Neuropsychiatry. 2017;07:509–521.

[62] Frank S, Copanaki E, Burbach GJ, Müller UC, Deller T. Differential regulation of toll-like receptor mRNAs in amyloid plaque-associated brain tissue of aged APP23 Transgenic mice. Neurosci Lett. 2009;453:41–4. 19429012 10.1016/j.neulet.2009.01.075

[63] Kumar A, Su Y, Sharma M, Singh S, Kim S, Peavey JJ, Suerken CK, Lockhart SN, Whitlow CT, Craft S, Hughes TM, Deep G. MicroRNA expression in extracellular vesicles as a novel blood-based biomarker for alzheimer’s disease. Alzheimers Dement. 2023;19:4952–66. 37071449 10.1002/alz.13055 PMC11663460

[64] Fabbri M, Paone A, Calore F, Galli R, Gaudio E, Santhanam R, Lovat F, Fadda P, Mao C, Nuovo GJ, Zanesi N, Crawford M, Ozer GH, Wernicke D, Alder H, Caligiuri MA, Nana-Sinkam P, Perrotti D, Croce CM. MicroRNAs bind to Toll-like receptors to induce prometastatic inflammatory response. Proc Natl Acad Sci U S A. 2012;109:E2110–2116. 22753494 10.1073/pnas.1209414109 PMC3412003

[65] Zhang A, Lu Y, Yuan L, Zhang P, Zou D, Wei F, Chen X. miR-29a-5p Alleviates Traumatic Brain Injury- (TBI-) Induced Permeability Disruption via Regulating NLRP3 Pathway. Dis Markers. 2021;2021:9556513. 10.1155/2021/9556513 PMC8645411 34876932

[66] Yang J-C, Zhao J, Chen Y-H, Wang R, Rong Z, Wang S-Y, Wu Y-M, Wang H-N, Yang L, Liu R. miR-29a-5p rescues depressive-like behaviors in a CUMS-induced mouse model by facilitating microglia M2-polarization in the prefrontal cortex via TMEM33 suppression. J Affect Disord. 2024;360:188–97. 38821373 10.1016/j.jad.2024.05.156

[67] Li Y, Zhou D, Ren Y, Zhang Z, Guo X, Ma M, Xue Z, Lv J, Liu H, Xi Q, Jia L, Zhang L, Liu Y, Zhang Q, Yan J, Da Y, Gao F, Yue J, Yao Z, Zhang R. Mir223 restrains autophagy and promotes CNS inflammation by targeting ATG16L1. Autophagy. 2018;15:478–92. 30208760 10.1080/15548627.2018.1522467 PMC6351131

[68] Wu C, Xing W, Zhang Y, Wang J, Zuo N, Sun F, Liu Q, Liu S. NLRP3/miR-223-3p axis attenuates neuroinflammation induced by chronic intermittent hypoxia. Funct Integr Genomics. 2023;23:342. 37991531 10.1007/s10142-023-01268-w

[69] Dowdy SF. Endosomal escape of RNA therapeutics: how do we solve this rate-limiting problem? RNA. 2023;29:396–401. 36669888 10.1261/rna.079507.122 PMC10019367

[70] Li Y-B, Fu Q, Guo M, Du Y, Chen Y, Cheng Y. MicroRNAs: pioneering regulators in alzheimer’s disease pathogenesis, diagnosis, and therapy. Transl Psychiatry. 2024;14:367. 39256358 10.1038/s41398-024-03075-8 PMC11387755

[71] Chen C-Y, Shih Y-C, Hung Y-F, Hsueh Y-P. Beyond defense: regulation of neuronal morphogenesis and brain functions via Toll-like receptors. J Biomed Sci. 2019;26:90. 31684953 10.1186/s12929-019-0584-z PMC6827257

[72] Zhang Z, Ohto U, Shibata T, Krayukhina E, Taoka M, Yamauchi Y, Tanji H, Isobe T, Uchiyama S, Miyake K, Shimizu T. Structural analysis reveals that Toll-like receptor 7 is a dual receptor for Guanosine and Single-Stranded RNA. Immunity. 2016;45:737–48. 27742543 10.1016/j.immuni.2016.09.011

[73] Zhang Z, Ohto U, Shibata T, Taoka M, Yamauchi Y, Sato R, Shukla NM, David SA, Isobe T, Miyake K, Shimizu T. Structural analyses of Toll-like receptor 7 reveal detailed RNA sequence specificity and recognition mechanism of agonistic ligands. Cell Rep. 2018;25:3371–e33815. 30566863 10.1016/j.celrep.2018.11.081

[74] Hu L, Si L, Dai X, Dong H, Ma Z, Sun Z, Li N, Sha H, Chen Y, Qian Y, Zhang Z. Exosomal miR-409-3p secreted from activated mast cells promotes microglial migration, activation and neuroinflammation by targeting Nr4a2 to activate the NF-κB pathway. J Neuroinflammation. 2021;18:68. 33750404 10.1186/s12974-021-02110-5 PMC7945321

[75] Long X, Yao X, Jiang Q, Yang Y, He X, Tian W, Zhao K, Zhang H. Astrocyte-derived exosomes enriched with miR-873a-5p inhibit neuroinflammation via microglia phenotype modulation after traumatic brain injury. J Neuroinflammation. 2020;17:89. 32192523 10.1186/s12974-020-01761-0 PMC7082961

[76] Browne TC, McQuillan K, McManus RM, O’Reilly J-A, Mills KHG, Lynch MA. IFN-γ production by amyloid β-specific Th1 cells promotes microglial activation and increases plaque burden in a mouse model of alzheimer’s disease. J Immunol. 2013;190:2241–51. 23365075 10.4049/jimmunol.1200947

[77] Altendorfer B, Unger MS, Poupardin R, Hoog A, Asslaber D, Gratz IK, Mrowetz H, Benedetti A, de Sousa DMB, Greil R, Egle A, Gate D, Wyss-Coray T, Aigner L. Transcriptomic profiling identifies CD8 + T cells in the brain of aged and alzheimer’s disease Transgenic mice as Tissue-Resident memory T cells. J Immunol. 2022;209:1272–85. 36165202 10.4049/jimmunol.2100737 PMC9515311

[78] Schneeberger S, Kim SJ, Geesdorf MN, Friebel E, Eede P, Jendrach M, Boltengagen A, Braeuning C, Ruhwedel T, Hülsmeier AJ, Gimber N, Foerster M, Obst J, Andreadou M, Mundt S, Schmoranzer J, Prokop S, Kessler W, Kuhlmann T, Möbius W, Nave K-A, Hornemann T, Becher B, Edgar JM, Karaiskos N, Kocks C, Rajewsky N, Heppner FL. Interleukin-12 signaling drives alzheimer’s disease pathology through disrupting neuronal and oligodendrocyte homeostasis. Nat Aging. 2025;5:622–41. 40082619 10.1038/s43587-025-00816-2 PMC12003168

[79] Lopez-Rodriguez AB, Hennessy E, Murray CL, Nazmi A, Delaney HJ, Healy D, Fagan SG, Rooney M, Stewart E, Lewis A, de Barra N, Scarry P, Riggs-Miller L, Boche D, Cunningham MO, Cunningham C. Acute systemic inflammation exacerbates neuroinflammation in alzheimer’s disease: IL-1β drives amplified responses in primed astrocytes and neuronal network dysfunction. Alzheimer’s Dement. 2021;17:1735–55. 34080771 10.1002/alz.12341 PMC8874214

[80] Hoye ML, Koval ED, Wegener AJ, Hyman TS, Yang C, O’Brien DR, Miller RL, Cole T, Schoch KM, Shen T, Kunikata T, Richard J-P, Gutmann DH, Maragakis NJ, Kordasiewicz HB, Dougherty JD, Miller TM. MicroRNA profiling reveals marker of motor neuron disease in ALS models. J Neurosci. 2017;37:5574–86. 28416596 10.1523/JNEUROSCI.3582-16.2017 PMC5452343

[81] Pomper N, Liu Y, Hoye ML, Dougherty JD, Miller TM. CNS MicroRNA profiles: a database for cell type enriched MicroRNA expression across the mouse central nervous system. Sci Rep. 2020;10:1–8. 32188880 10.1038/s41598-020-61307-5 PMC7080788

[82] Kim EK, Choi E-J. Pathological roles of MAPK signaling pathways in human diseases. Biochim Biophys Acta. 2010;1802:396–405. 20079433 10.1016/j.bbadis.2009.12.009

[83] Detka J, Płachtij N, Strzelec M, Manik A, Sałat K. p38α Mitogen-Activated protein Kinase—An emerging drug target for the treatment of alzheimer’s disease. Molecules. 2024;29:4354. 39339348 10.3390/molecules29184354 PMC11433989

[84] An N, Bassil K, Al Jowf GI, Steinbusch HWM, Rothermel M, de Nijs L, Rutten BPF. Dual-specificity phosphatases in mental and neurological disorders. Prog Neurobiol. 2021;198:101906. 32905807 10.1016/j.pneurobio.2020.101906

[85] Du Y, Du Y, Zhang Y, Huang Z, Fu M, Li J, Pang Y, Lei P, Wang YT, Song W, He G, Dong Z. MKP-1 reduces Aβ generation and alleviates cognitive impairments in alzheimer’s disease models. Sig Transduct Target Ther. 2019;4:1–12. 10.1038/s41392-019-0091-4 PMC6895219 31840000

[86] Banzhaf-Strathmann J, Benito E, May S, Arzberger T, Tahirovic S, Kretzschmar H, Fischer A, Edbauer D. MicroRNA-125b induces Tau hyperphosphorylation and cognitive deficits in alzheimer’s disease. EMBO J. 2014;33:1667–80. 25001178 10.15252/embj.201387576 PMC4194100

[87] Peroval MY, Boyd AC, Young JR, Smith AL. A critical role for MAPK signalling pathways in the transcriptional regulation of toll like receptors. PLoS ONE. 2013;8:e51243. 23405061 10.1371/journal.pone.0051243 PMC3566169

[88] Oda K, Kitano H. A comprehensive map of the toll-like receptor signaling network. Mol Syst Biol. 2006;2:2006.0015. 10.1038/msb4100057 PMC1681489 16738560

[89] Bachstetter AD, Xing B, de Almeida L, Dimayuga ER, Watterson DM, Van Eldik LJ. Microglial p38α MAPK is a key regulator of Proinflammatory cytokine up-regulation induced by toll-like receptor (TLR) ligands or beta-amyloid (Aβ). J Neuroinflammation. 2011;8:79. 21733175 10.1186/1742-2094-8-79 PMC3142505

[90] Rosi S. Neuroinflammation and the plasticity-related immediate-early gene Arc. Brain Behav Immun. 2011;25:S39–49. 21320587 10.1016/j.bbi.2011.02.003 PMC3098296

[91] Hung Y-F, Chen C-Y, Shih Y-C, Liu H-Y, Huang C-M, Hsueh Y-P. Endosomal TLR3, TLR7, and TLR8 control neuronal morphology through different transcriptional programs. J Cell Biol. 2018;217:2727–42. 29777026 10.1083/jcb.201712113 PMC6080926

[92] Rothe T, Ipseiz N, Faas M, Lang S, Perez-Branguli F, Metzger D, Ichinose H, Winner B, Schett G, Krönke G. The nuclear receptor Nr4a1 acts as a microglia rheostat and serves as a therapeutic target in Autoimmune-Driven central nervous system inflammation. J Immunol. 2017;198:3878–85. 28411187 10.4049/jimmunol.1600638 PMC5798579

[93] Saijo K, Winner B, Carson CT, Collier JG, Boyer L, Rosenfeld MG, Gage FH, Glass CK. A Nurr1/CoREST transrepression pathway attenuates neurotoxic inflammation in activated microglia and astrocytes. Cell. 2009;137:47–59. 19345186 10.1016/j.cell.2009.01.038 PMC2754279

[94] Yang G, Pan Q, Lu Y, Zhu J, Gou X. miR-29a-5p modulates ferroptosis by targeting ferritin heavy chain FTH1 in prostate cancer. Biochem Biophys Res Commun. 2023;652:6–13. 36806086 10.1016/j.bbrc.2023.02.030

[95] Li W, Jiang Y, Pan Q, Yang G. miR-29a-5p regulates the malignant biological process of liver cancer cells through ARID2 regulation of EMT. Adv Clin Exp Med. 2023;32:575–82. 36530029 10.17219/acem/156646

[96] Dai Y, Chen Z, Zhao W, Cai G, Wang Z, Wang X, Hu H, Zhang Y. miR-29a-5p regulates the Proliferation, Invasion, and migration of gliomas by targeting DHRS4. Front Oncol. 2020;10:1772. 33014873 10.3389/fonc.2020.01772 PMC7511594

[97] Hébert SS, Horré K, Nicolaï L, Papadopoulou AS, Mandemakers W, Silahtaroglu AN, Kauppinen S, Delacourte A, De Strooper B. Loss of MicroRNA cluster miR-29a/b-1 in sporadic alzheimer’s disease correlates with increased BACE1/beta-secretase expression. Proc Natl Acad Sci U S A. 2008;105:6415–20. 18434550 10.1073/pnas.0710263105 PMC2359789

[98] Peña-Bautista C, Tarazona-Sánchez A, Braza-Boils A, Balaguer A, Ferré-González L, Cañada-Martínez AJ, Baquero M, Cháfer-Pericás C. Plasma MicroRNAs as potential biomarkers in early alzheimer disease expression. Sci Rep. 2022;12:15589. 36114255 10.1038/s41598-022-19862-6 PMC9481579

[99] Geekiyanage H, Jicha GA, Nelson PT, Chan C. Blood serum mirna: non-invasive biomarkers for alzheimer’s disease. Exp Neurol. 2012;235:491–6. 22155483 10.1016/j.expneurol.2011.11.026 PMC3361462

[100] Ramirez-Gomez J, Dalal S, Devara D, Sharma B, Rodarte D, Kumar S. MicroRNA-based recent research developments in alzheimer’s disease. J Alzheimers Dis. 2025;104:14–31. 39894921 10.1177/13872877241313397

[101] Müller M, Jäkel L, Bruinsma IB, Claassen JA, Kuiperij HB, Verbeek MM. MicroRNA-29a is a candidate biomarker for alzheimer’s disease in Cell-Free cerebrospinal fluid. Mol Neurobiol. 2016;53:2894–9. 25895659 10.1007/s12035-015-9156-8 PMC4902829

[102] Li Y, Tan J, Miao Y, Zhang Q. MicroRNA in extracellular vesicles regulates inflammation through macrophages under hypoxia. Cell Death Discov. 2021;7:1–12. 10.1038/s41420-021-00670-2 PMC8505641 34635652

