# Supplementary material for: MicroRNA-29a-5p contributes to neuroinflammation through TLR7
Source: J Neuroinflammation. 2026 Jan 9;23:38. doi: 10.1186/s12974-025-03680-4 (PMC12849500; doi:10.1186/s12974-025-03680-4)
Supplement: Supplementary file 1 — Supplementary Material 1. Figure S1. LyoVec transfection increases microglial TNF response to miR-29a-5p, and miR-29a-5p co-localises to endosomes in microglia. A) C57BL/6 (WT) microglia were exposed to free or LyoVec-transfected miR-29a-5p and mutant oligonucleotide (10 μg/mL) for 24 h. TNF concentration in the supernatant was subsequently assessed by ELISA. LPS (100 ng/mL) served as a positive control (n = 3). B) WT microglia were incubated with pHrodo Red (20 μg/mL) and Alexa-488-conjugated miR-29a-5p (10 μg/mL) for 4 h with line profiles depicting fluorescence intensity in marked region of interest (white line). Figure S2. Extracellular miR-29a-5p induces transcriptional TLR pathway changes in microglia in vitro. A) WT and Tlr7−/− microglia were exposed to LPS (100 ng/mL) for 24 h. B) WT microglia were exposed to LPS (100 ng/mL) or miR-29a-5p (10 μg/mL) for 6 h. C) WT neurons were exposed to miR-29a-5p (10 μg/mL) for 48 h. D) miR-29a-5p expression was assessed in WT microglia exposed to miR-29a-5p (10 μg/mL), Aβ (10 μM), or loxoribine (1 mM) for 24 h. For all experiments, RNA was extracted from cells for analysis by RT-qPCR. Significance tested by multiple unpaired t-tests by two-stage step-up method A-C) or one-way ANOVA followed by Sidak’s multiple comparison test D). **p < 0.01 to unstimulated, †p < 0.05 to gene of interest exposed to different conditions. Dashed line represents unstimulated condition. Error bars represent mean ± SEM. n = 3. Figure S3. Effect of extracellular miR-29a-5p on cortical neurons in vitro. A) Representative images of NeuN and Iba1 immunostaining after 5 d in co-cultures of WT neurons and WT microglia exposed to mut. oligo, miR-29a-5p (10 μg/mL), or loxoribine (1 mM). Scale bar represents 50 μM. B, C) Cultures of WT microglia were stimulated with mut. oligo, miR-29a-5p (10 μg/mL), or loxoribine (1 mM) for 24 h to create conditioned media. Cultures of enriched cortical neurons were exposed to this microglia-conditioned media for 5 d. [file 12974_2025_3680_MOESM1_ESM.pdf]

Fig. S1

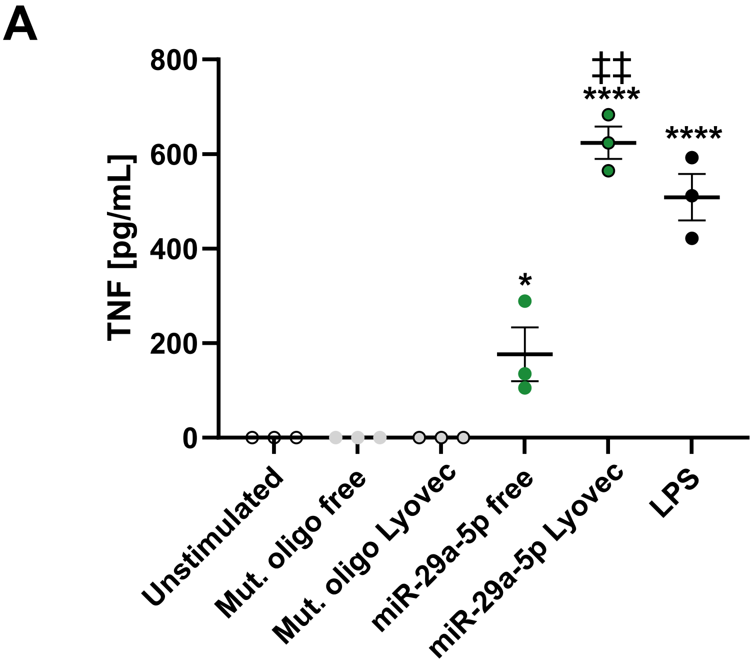
A
800
600
400
200
0
TNF [pg/mL]
‡‡
****
****
*
Unstimulated
Mut. oligo free
Mut. oligo Lyovec
miR-29a-5p free
miR-29a-5p Lyovec
LPS

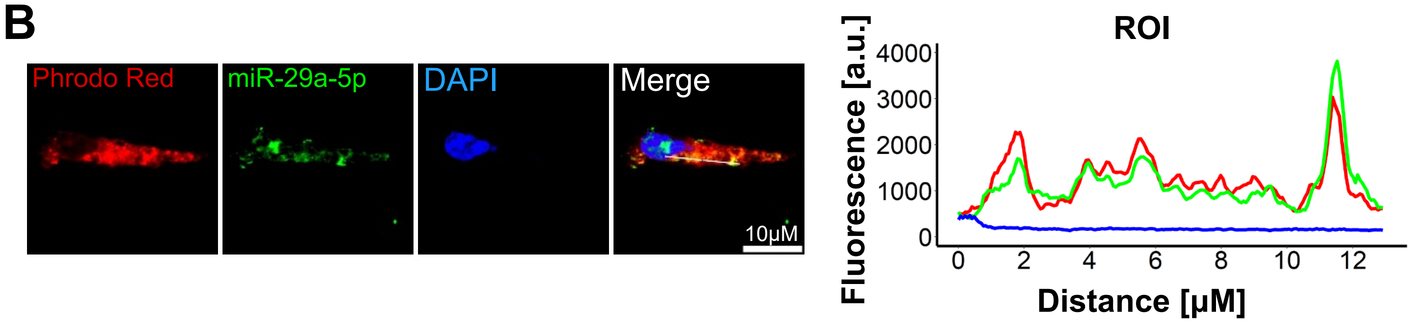
B
Phrodo Red
miR-29a-5p
DAPI
Merge
10µM
ROI
Fluorescence [a.u.]
4000
3000
2000
1000
0
0
2
4
6
8
10
12
Distance [µM]

Fig. S2

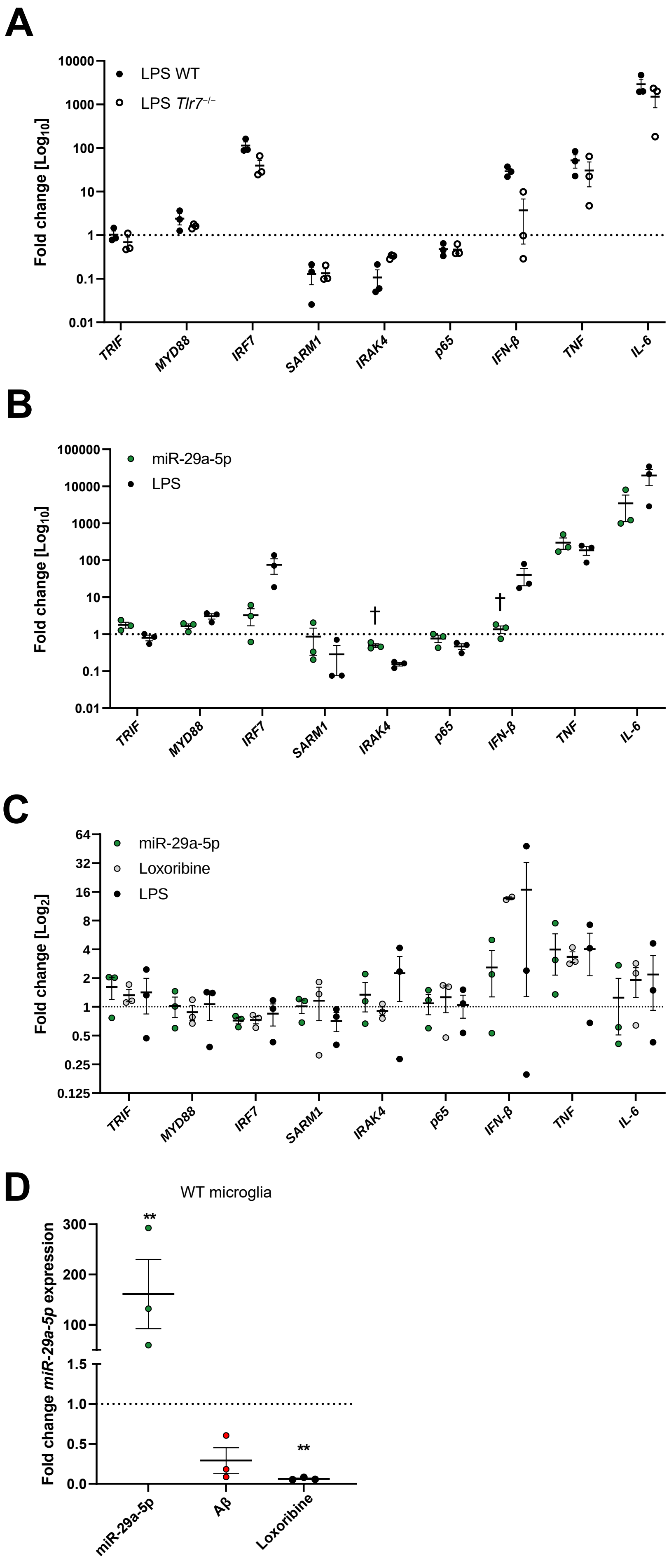

A
LPS WT
LPS Tlr7−/−
Fold change [Log10]
TRIF
MYD88
IRF7
SARM1
IRAK4
p65
IFN-β
TNF
IL-6
B
miR-29a-5p
LPS
Fold change [Log10]
C
miR-29a-5p
Loxoribine
LPS
Fold change [Log2]
D
WT microglia
Fold change miR-29a-5p expression
miR-29a-5p
Aβ
Loxoribine

Fig. S3

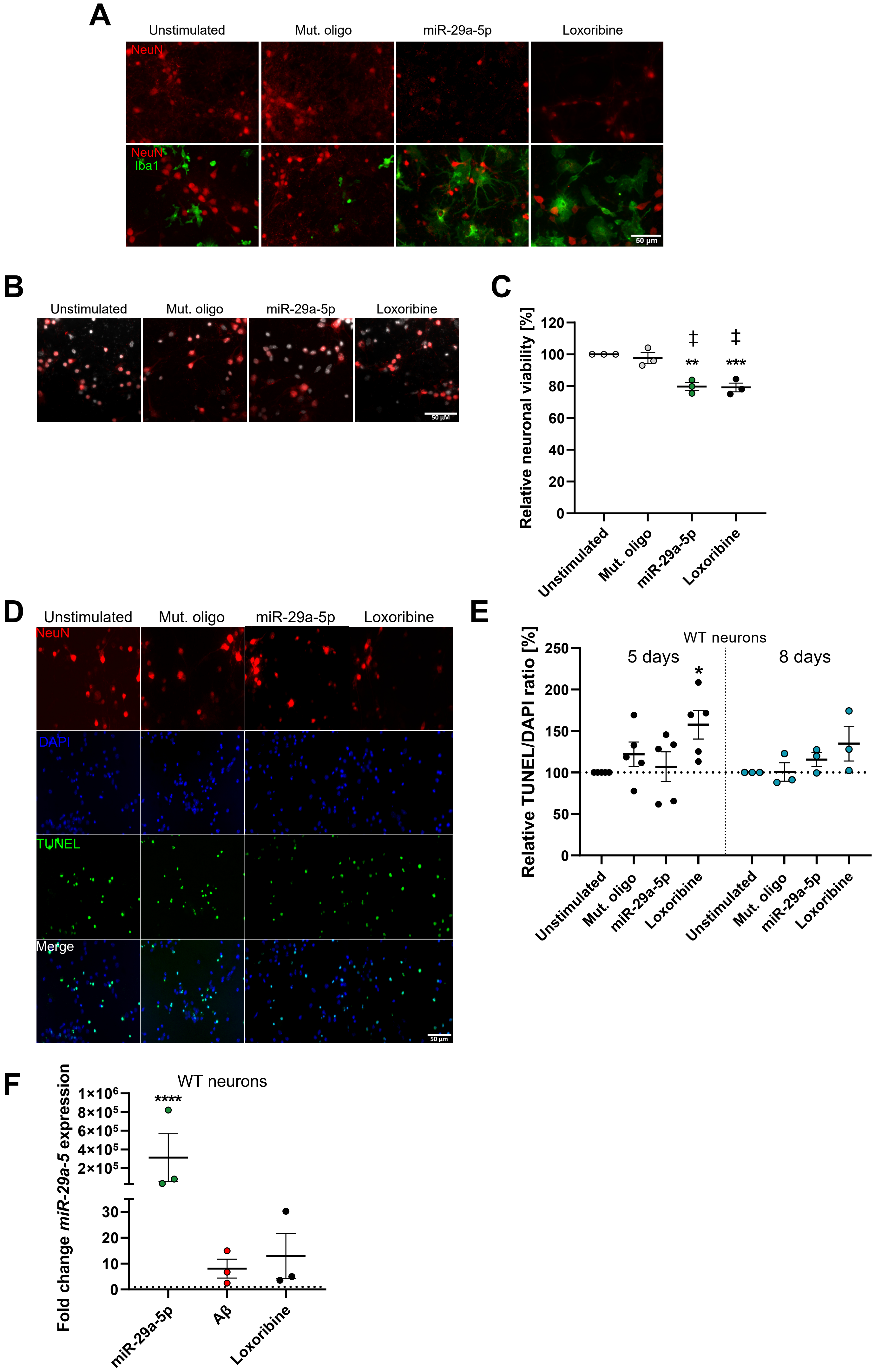
A
Unstimulated
Mut. oligo
miR-29a-5p
Loxoribine
NeuN
NeuN
Iba1
50 µm
B
Unstimulated
Mut. oligo
miR-29a-5p
Loxoribine
50 µM
C
Relative neuronal viability [%]
120
100
80
60
40
20
0
‡
‡
**
***
Unstimulated
Mut. oligo
miR-29a-5p
Loxoribine
D
Unstimulated
Mut. oligo
miR-29a-5p
Loxoribine
NeuN
DAPI
TUNEL
Merge
50 µm
E
WT neurons
5 days
8 days
*
Relative TUNEL/DAPI ratio [%]
250
200
150
100
50
0
Unstimulated
Mut. oligo
miR-29a-5p
Loxoribine
Unstimulated
Mut. oligo
miR-29a-5p
Loxoribine
F
WT neurons
****
Fold change miR-29a-5 expression
1×10^6
8×10^5
6×10^5
4×10^5
2×10^5
30
20
10
0
miR-29a-5p
Aβ
Loxoribine

Fig. S4

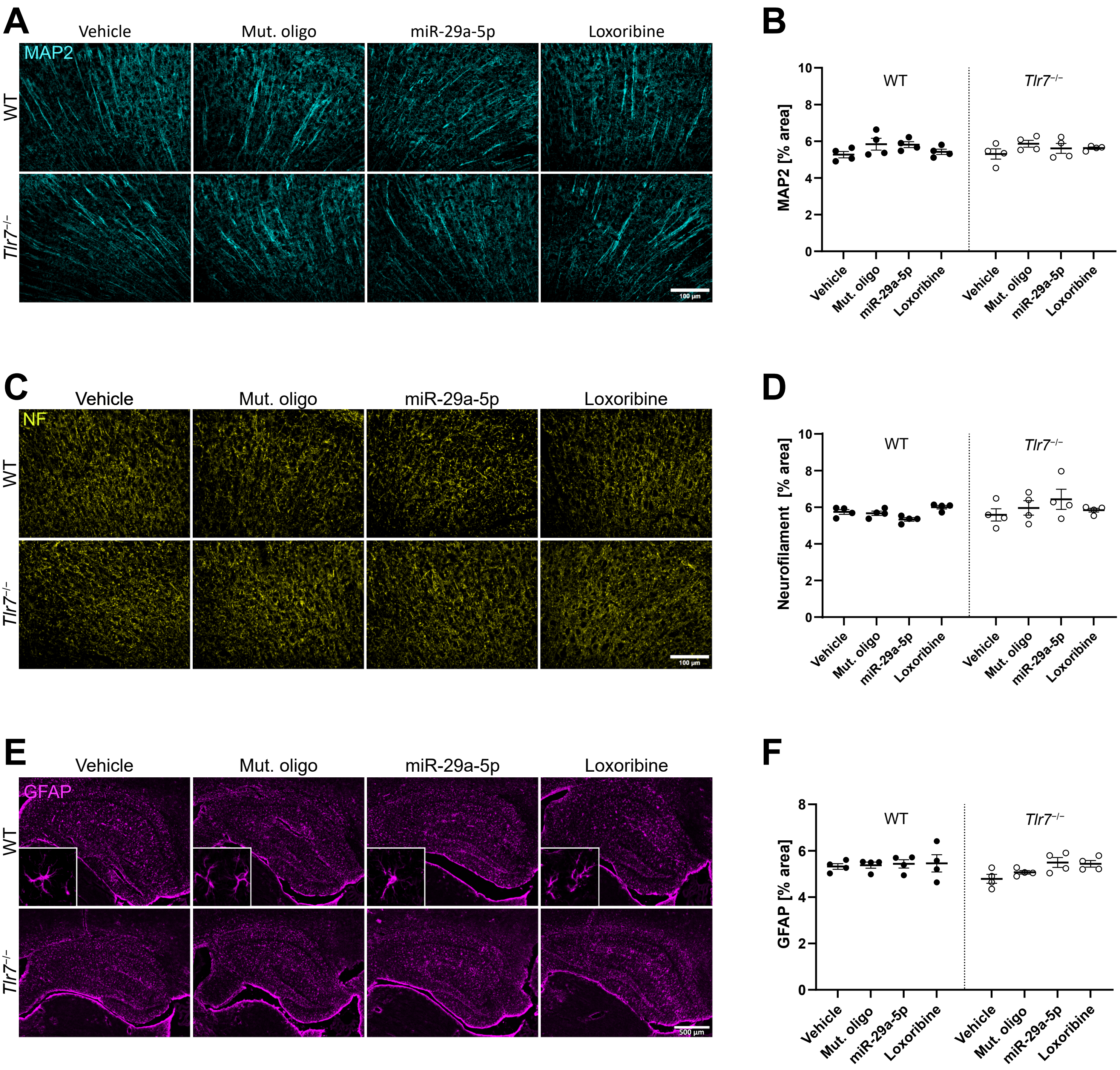

A
Vehicle
Mut. oligo
miR-29a-5p
Loxoribine
MAP2
WT
Tlr7−/−
100 µm
B
MAP2 [% area]
WT
Tlr7−/−
C
NF
100 µm
D
Neurofilament [% area]
E
GFAP
500 µm
F
GFAP [% area]

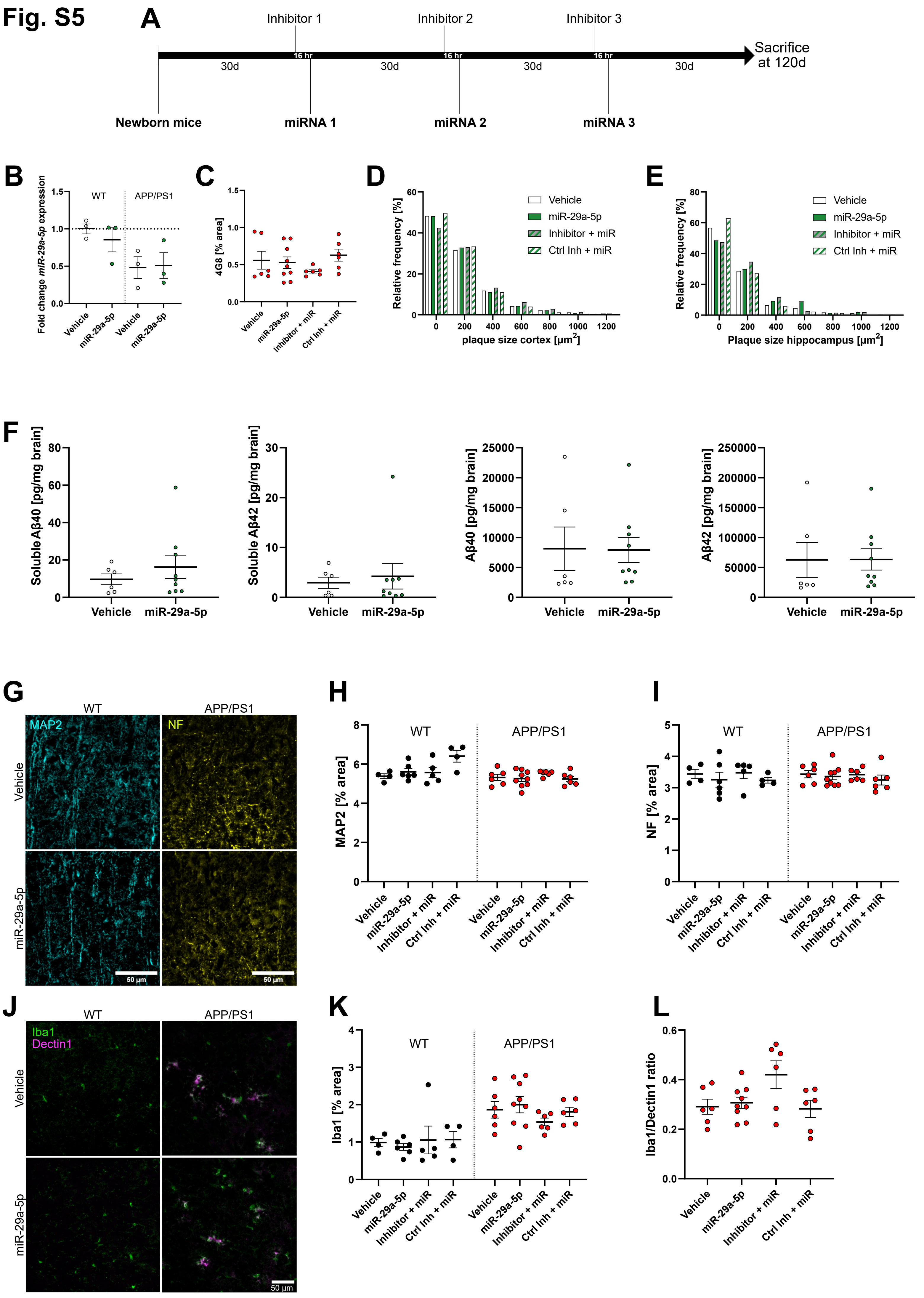
Fig. S5
A
Inhibitor 1
Inhibitor 2
Inhibitor 3
16 hr
30d
Sacrifice at 120d
Newborn mice
miRNA 1
miRNA 2
miRNA 3
B
WT
APP/PS1
Fold change miR-29a-5p expression
Vehicle
miR-29a-5p
C
4G8 [% area]
Inhibitor + miR
Ctrl Inh + miR
D
Relative frequency [%]
plaque size cortex [µm2]
E
Plaque size hippocampus [µm2]
F
Soluble Aβ40 [pg/mg brain]
Soluble Aβ42 [pg/mg brain]
Aβ40 [pg/mg brain]
Aβ42 [pg/mg brain]
G
MAP2
NF
50 µm
H
MAP2 [% area]
I
NF [% area]
J
Iba1
Dectin1
K
Iba1 [% area]
L
Iba1/Dectin1 ratio

Fig. S6

A

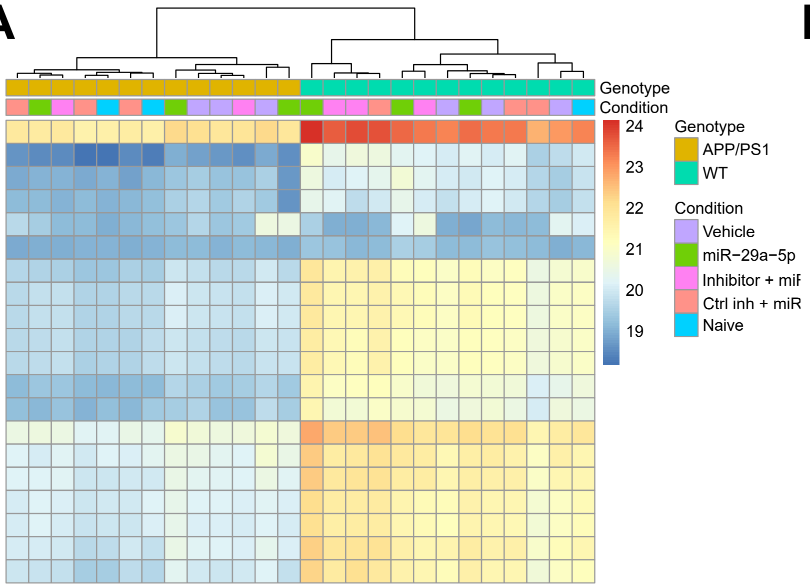

B

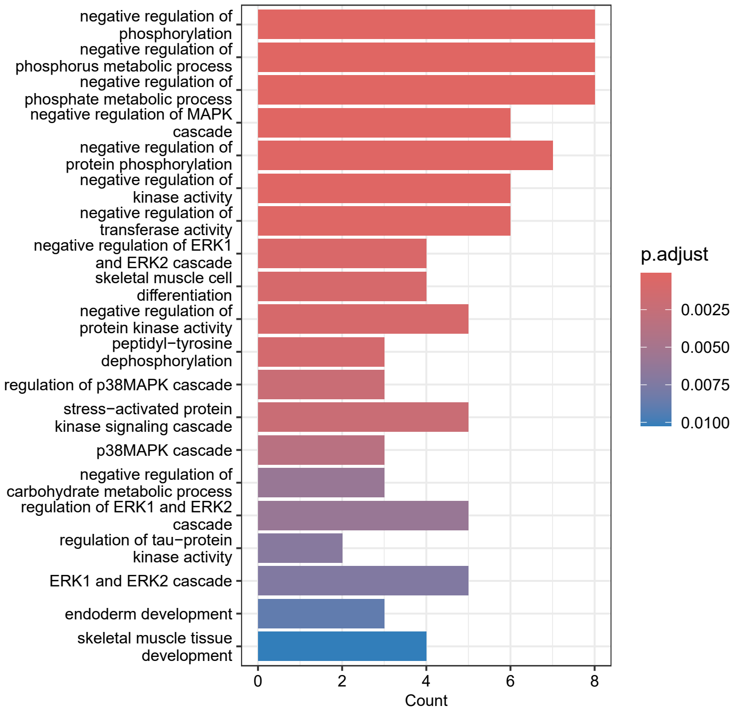

C

Hallmark pathway enrichment (MSigDB)	NES	P value	Gene list
TNFA_SIGNALING_VIA_NFKB	-1.57919	0.00181	Fos, Tiparp, Dusp1, Junb, Nr4a1, Gadd45a, Dusp5, Trib1, Trib1, Egr1, Egr2, Ier2, Per1

D

Database	# potential target genes	WT			APP/PS1			Combined		
		miR-29a-5p	Inh + miR	Ctrl inh + miR	miR-29a-5p	Inh + miR	Ctrl inh + miR	miR-29a-5p	Inh + miR	Ctrl inh + miR
miRDB	651	0	1	0	0	1	6	0	8	8
			Lrrc34			Hephl1	NR4A3, Acvr2a, Ano5, Arhgap6, Npy2r, Tacr3		Kcna1, Csmd1, Cenpf, Crbn, Dll4, Acvr2a, Purg, Npy2r	Nr4a3, DLL4, Phrmc1, Acvr2a, Mgat4c, Robo1, Arhgap6, Npy2r
miRtarBase	94	0	1	0	0	0	0	0	3	2
			PAQR3						Ptprt, Paqr3 ,Gem	Srpx2, Sowahb
